# Transcriptomic FHIT^low^/pHER2^high^ signature as a predictive factor of outcome and immunotherapy response in non-small cell lung cancer

**DOI:** 10.3389/fimmu.2022.1058531

**Published:** 2022-12-05

**Authors:** Audrey Brisebarre, Julien Ancel, Théophile Ponchel, Emma Loeffler, Adeline Germain, Véronique Dalstein, Valérian Dormoy, Anne Durlach, Gonzague Delepine, Gaëtan Deslée, Myriam Polette, Béatrice Nawrocki-Raby

**Affiliations:** ^1^ INSERM, Université de Reims Champagne-Ardenne, P3Cell, UMR-S 1250, SFR CAP Santé, Reims, France; ^2^ CHU Reims, Hôpital Maison Blanche, Service de Pneumologie, Reims, France; ^3^ CHU Reims, Pôle de Biologie Territoriale, Service de Pathologie, Reims, France; ^4^ CHU Reims, Hôpital Robert Debré, Service de Chirurgie cardio-vasculaire et thoracique, Reims, France

**Keywords:** NSCLC, FHIT, HER2, transcriptomic signature, prognosis, immunotherapy response

## Abstract

**Introduction:**

In recent decades, the development of immunotherapy and targeted therapies has considerably improved the outcome of non-small cell lung cancer (NSCLC) patients. Despite these impressive clinical benefits, new biomarkers are needed for an accurate stratification of NSCLC patients and a more personalized management. We recently showed that the tumor suppressor fragile histidine triad (FHIT), frequently lost in NSCLC, controls HER2 receptor activity in lung tumor cells and that tumor cells from NSCLC patients harboring a FHIT^low^/pHER2^high^ phenotype are sensitive to anti-HER2 drugs. Here, we sought to identify the transcriptomic signature of this phenotype and evaluate its clinical significance.

**Materials and methods:**

We performed RNA sequencing analysis on tumor cells isolated from NSCLC (n=12) according to FHIT/pHER2 status and a functional analysis of differentially regulated genes. We also investigated the FHIT^low^/pHER2^high^ signature in The Cancer Genome Atlas (TCGA) lung adenocarcinoma (LUAD) (n=489) and lung squamous cell carcinoma (LUSC) (n=493) cohorts and used the tumor immune dysfunction and exclusion (TIDE) model to test the ability of this signature to predict response to immune checkpoint inhibitors (ICI).

**Results:**

We showed that up-regulated genes in FHIT^low^/pHER2^high^ tumors were associated with cell proliferation, metabolism and metastasis, whereas down-regulated genes were related to immune response. The FHIT^low^/pHER2^high^ signature was associated with the higher size of tumors, lymph node involvement, and late TNM stages in LUAD and LUSC cohorts. It was identified as an independent predictor of overall survival (OS) in LUAD cohort. FHIT^low^/pHER2^high^ tumors were also predictive of poor response to ICI in both LUAD and LUSC cohorts.

**Conclusion:**

These data suggest that ICI might not be a relevant option for NSCLC patients with FHIT^low^/pHER2^high^ tumors and that anti-HER2 targeted therapy could be a good therapeutic alternative for this molecular subclass with poorer prognosis.

## 1 Introduction

Lung cancer is a leading cause of death worldwide. About 2 million cases are diagnosed each year causing 1.76 million deaths (1). In recent decades, major advances have been achieved in the treatment of non-small cell lung cancer (NSCLC), the most widespread lung cancer, with the development of immunotherapy and targeted therapies according to oncogenic driver alterations. Impressive clinical benefits have been obtained with immune checkpoint inhibitors (ICI) (2). Despite the substantial improvement of prognosis, partly conditioned by programmed death-ligand 1 (PD-L1) expression level, some NSCLC patients may not respond to ICI (1–3). On another hand, current targeted drugs are approved for EGFR, ALK, ROS1, BRAF or NTRK molecular alterations (1, 4). In contrast to breast and gastric cancers, anti-HER2 treatments are not a standard of care for NSCLC management, despite recent promising results with HER2 antibody-drug conjugates such as trastuzumab-deruxtecan (5, 6).

The activation of HER2 in NSCLC is known to occur by three described mechanisms, including gene mutation, gene amplification and protein overexpression, which result in specific prognostic and predictive outcomes (5). We recently identified another mechanism of HER2 activation, assessed by HER2 phosphorylation (pHER2), and regulated by fragile histidine triad (FHIT), a tumor suppressor frequently lost in NSCLC (7, 8). FHIT controls HER2 receptor activity in lung tumor cells, and thereby, lung tumor cells with a loss of FHIT expression and consecutive activation of HER2 receptor are sensitive to anti-HER2 drugs. We previously proposed a new FHIT^low^/pHER2^high^ NSCLC phenotype that may be eligible for an HER2-targeted therapy (7). This phenotype represents about 25% of NSCLC independently of histological type and is associated with a poor degree of tumor differentiation (7).

With the aim of better characterizing the FHIT^low^/pHER2^high^ phenotype and its clinical significance, we investigated its associated-transcriptomic signature. We therefore performed RNA sequencing analysis on tumor cells isolated from NSCLC displaying or not a FHIT^low^/pHER2^high^ status and evaluated this new FHIT^low^/pHER2^high^ molecular subclass in TCGA NSCLC cohorts for both prognosis and ICI sensitivity.

## 2 Materials and methods

### 2.1 Ethics approval and consent to participate

Human study was conducted in accordance with the ethical guideline of the Declaration of Helsinki. Human tumors were obtained from the Tumor Bank of the Reims University Hospital Biological Resource Collection NO. AC-2019-340 declared at the Ministry of Health according to the French Law, for use of tissue samples for research. Surgically resected tumors were collected after obtaining informed consent from patients with NSCLC. Access to patient data for this non-interventional study was approved by the French national commission CNIL (Commission Nationale de l’Informatique et des Libertés) (NO.2049775 v 0).

### 2.2 Primary tumor cells

Primary tumor cells were obtained from 38 NSCLC fresh samples. Freshly resected tumors were cut into small pieces, then digested overnight at +4°C in a 0.1% Pronase E solution (Sigma-Aldrich, Saint-Louis, MO) and seeded in type IV collagen-coated dishes with CnT-17 medium (CELLnTEc, Bern, Switzerland). After the proliferation phase, cells were cultured in bronchial epithelial cell growth medium (BEGM) (Lonza, Walkersville, MD). The sensitivity to the HER2 tyrosine kinase inhibitor (TKI) tucatinib (irbinitinib, ARRY-380, ONT-380) (HY-16069, MedChemtronica, Sollentuna, Sweden) was assessed by MTT assay as previously described (7). Further culture samples were also frozen for protein extraction and RNA extraction. FHIT and pHER2 status were assessed by western blotting as previously described (**Supplementary Figure S1**) (7). Six FHIT^low^/pHER2^high^ tumors and 6 other tumors were selected for RNA-sequencing analysis (**Supplementary Figure S1** and **Supplementary Table S1**).

### 2.3 RNA isolation and library preparation

Total RNA was purified from frozen primary tumor cell pellets with RNeasy Plus Micro Kit (Qiagen, Hilden, Germany) according to the manufacturer’s specifications. During the procedure, contaminant DNA was eliminated on a gDNA Eliminator spin column. Total RNA concentration was determined by measuring the absorbance at 260 nm on a NanoDrop 1000 spectrophotometer (Thermo Scientific, Madison, WI, USA). The integrity and size distribution of purified total RNA were checked using the Experion RNA StdSens Analysis Kit on the Experion automated electrophoresis system (Bio-Rad, Hercules, CA, USA). Total RNA samples with a RQI>7 were send to Integragen (Evry, France) for libraries preparation using 400 ng. Libraries were prepared with NEBNext Ultra II Directional RNA Library Prep Kit for Illumina protocol according to supplier recommendations. Briefly the key stages of this protocol were successively, the purification of PolyA containing mRNA molecules using poly-T oligo attached magnetic beads from 100 ng total RNA (with the Magnetic mRNA Isolation Kit from NEB), a fragmentation using divalent cations under elevated temperature to obtain approximately 300 bp pieces, double-strand cDNA synthesis and finally Illumina adapters ligation and cDNA library amplification by PCR for sequencing.

### 2.4 Next-generation sequencing

Sequencing was then carried out by Integragen on Paired-End 100 b reads on Illumina NovaSeq in two different sequencing runs. Image analysis and base calling were performed using Illumina Real-Time Analysis (3.4.4) with default parameters.

### 2.5 Data analysis

#### 2.5.1 Differential gene expression

Quality control of raw sequence data was performed using FastQC (version 0.11.5) (9). Head bases were trimmed for adaptor sequences, and low-complexity or low-quality sequences were removed with Trimmomatic (version 0.39) (10). The remaining sequences were mapped to the Homo sapiens hg38 reference genome assembly (hg38.fa) using tophat2 (version 2.1.1) with stringent parameters generating bam format (11, 12). The quality of alignment was checked using metrics provided by qualimap (version 2.2.1) and low-quality alignments were removed (13). Raw counts were obtained using htseq-count (version 0.6.1) (14). Differential expression analysis was performed using DESeq2, an R package (15, 16). Raw counts were normalized using a scaling factor based on median gene expression across the samples and filtered to exclude genes with fewer than 5 counts across the samples. 15,955 genes were expressed with these parameters. A batch covariate was included in the design model to estimate the effect of a two-runs sequencing. Genes with a Benjamini-Hochberg FDR lower than 0.05 and a two-fold change in expression were considered as significantly differentially expressed. A volcano plot was also created to examine the distribution of log2 fold change at different significance levels.

#### 2.5.2 Functional analysis of the differentially expressed genes

To functionally describe the differentially expressed genes, the Gene Set Enrichment Analysis (GSEA version 4.1.0) was performed (17, 18). The 50 hallmark gene sets from MsigDB data base representing well-defined biological states and processes were tested for their association with the FHIT/pHER2 status (18, 19). Differentially expressed genes were analyzed in terms of biological processes (BP) from Gene Ontology resource (GOterms) with the ViSEAGO R package using the Ensembl data base option (15, 20–23). Briefly, genes were annotated and enriched GO terms were computed and clustered depending on their semantic similarity calculated from their information content using Lin distance. Semantic similarity between the clusters was calculated using the BMA algorithm and was used to perform a hierarchical clustering of the clusters with the war.D2 aggregation criteria. Differentially expressed genes were also mapped to Reactome pathways and over-representation was calculated with hypergeometric distribution corrected for FDR using the Benjamini-Hochberg method on their online platform (24). Dot plots were produced with the ggplot2 R package (15, 25).

#### 2.5.3 Test of the FHIT^low^/pHER2^high^ signature to predict NSCLC patient outcome and response to immunotherapy

For the following analyses, two NSCLC cohorts were used: the Firehose Legacy TCGA lung adenocarcinoma (LUAD) cohort and the Firehose Legacy TCGA lung squamous cell carcinoma (LUSC) cohort downloaded from the cBioportal (26, 27). Patients with metastatic tumors were excluded from the study thus resulting in 489 patients analyzed for LUAD and 493 patients analyzed for LUSC. A score for each patient based on the expression values of the 983 differentially expressed genes (sum of the expressions of genes up-regulated – sum of the expressions of genes down-regulated) was calculated to determine the FHIT/pHER2 status of the TCGA tumors. For each cohort, we defined FHIT^low^/pHER2^high^ tumors as those with a score superior to the third quartile and the other tumors as those with a score inferior to the first quartile. Clinical and survival data were extracted for these tumors. The TIDE (Tumor Immune Dysfunction and Exclusion) online module was also used to calculate a score based on the gene expression profile of each tumor and to predict patient response to immunotherapies (28, 29).

### 2.6 Statistics

Associations between clinical parameters and FHIT/pHER2 status were analyzed with the two-tailed Mann-Whitney test (age) or the two-sided Fisher’s exact test (sex, tumor size, lymph node status, tumor node metastasis (TNM) stage) using R or Prism version 5.0 software (GraphPad Software, La Jolla, CA) (15). An estimate of the survival curve for censored data using the Kaplan-Meier method was computed using the surfit function of the survival R package (15, 30). The curves were plotted with the ggsurvplot function of the survminer R package (15, 31). P-values of the log-rank test were calculated to test the difference between the two curves. A Cox proportional hazards regression model was estimated independently for each variate known to potentially influence survival in patients with NSCLC (age, sex, and TNM stage) and for the FHIT/pHER2 status with the coxph function of the survival R package (15, 30, 32). The significant variates were included in a multivariate Cox proportional hazards regression model. Hazard Risks (HR) were calculated as the exponential of the model’s coefficients. The waterfall plots of the TIDE scores were plotted with ggplot function (25). P < 0.05 was considered significant.

## 3 Results

### 3.1 Transcriptomic signature of FHIT^low^/pHER2^high^ tumors

**Figure 1:**
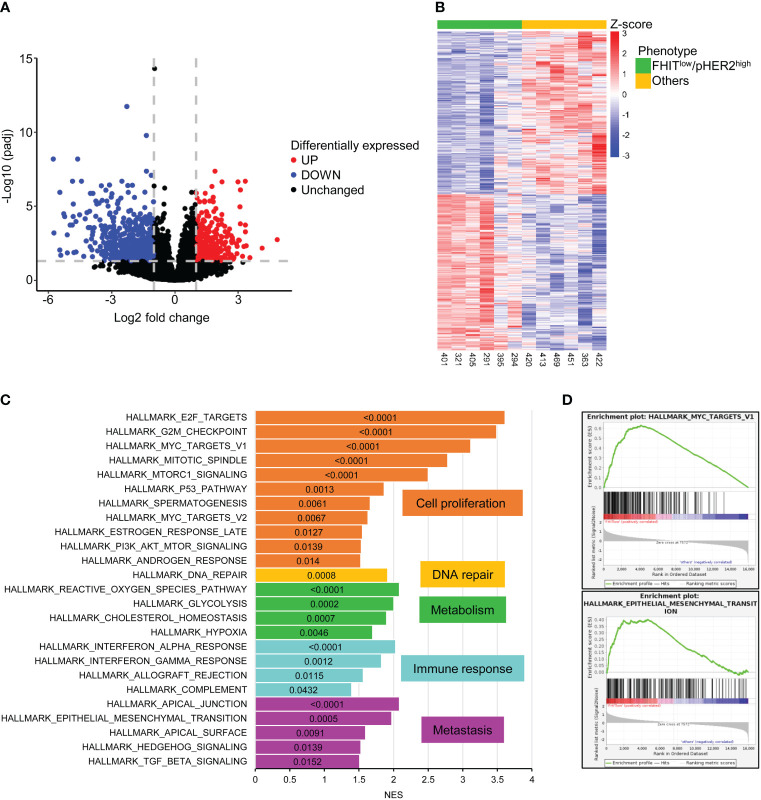
983 genes are significantly differentially expressed between FHIT^low^/pHER2^high^ and other tumors. **(A)** Volcano plot of statistical significance against fold-change in log2 scale between FHIT^low^/pHER2^high^ and other tumors highlighting the significantly downregulated genes in FHIT^low^/pHER2^high^ tumors in blue and the significantly upregulated genes in red. The gray horizontal dashed line corresponds to a False Discovery Rate (FDR) of 0.05 and the two vertical gray dashed lines correspond to a fold change of 0.5 and 2. **(B)** Heatmap of differentially expressed mRNAs. Scaled rlog transformed values are plotted in FHIT^low^/pHER2^high^ (green) against other (yellow) tumors. Each column represents a tumor, whereas each line represents a gene. Expression values by row (by gene) are centered so that the color reflects the amount by which each gene deviates in a specific sample from the gene’s average across all samples. The tumors are clustered in an unsupervised hierarchical way. The top of the graph shows the sample’s membership. **(C, D)** Gene Set Enrichment Analysis (GSEA) was performed on all cancer hallmarks referenced in Msig database. Bar plot of the cancer hallmarks enriched in FHIT^low^/pHER2^high^ tumors **(C)**. Normalized Enrichment Score (NES) was plotted on the abscissa and the False Discovery Rate (FDR) corresponding to each enrichment test was written inside the bar of the corresponding hallmark. FDR < 0.05 was considered statistically significant. Hallmarks are grouped and colored depending on the cancer process they belong to. Enrichment of hallmarks of MYC targets V1 and Epithelial to Mesenchymal Transition (EMT) in FHIT^low^/pHER2^high^ tumors are shown **(D)**.

To define the transcriptomic signature associated with the FHIT^low^/pHER2^high^ phenotype, we selected 6 FHIT^low^/pHER2^high^ cases and 6 other cases in a cohort of primary tumor cells from NSCLC patients whose FHIT and pHER2 status were assessed by western blotting (**Supplementary Figure S1**). Each group contained 4 adenocarcinomas (ADC) and 2 squamous cell carcinomas (SCC). FHIT^low^/pHER2^high^ tumors were confirmed to be more sensitive to the TKI tucatinib (**Supplementary Table S1**). RNA samples corresponding to these cases were subjected to a RNA-sequencing analysis (**Supplementary Figure S2**). Nine hundred and eighty three genes were found significantly differentially expressed between FHIT^low^/pHER2^high^ and other tumors (**Figure 1A**, **Supplementary Table S2**) and their expression allowed to separate the two groups of phenotype (**Figure 1B**). Among them, 620 genes were down-regulated and 363 genes up-regulated in FHIT^low^/pHER2^high^ group. Thirty one down-regulated genes are known to be prognostic markers of lung cancer, 28 of which are of favorable prognosis, such as GDPD1, SLC46A3, CLIC6, LZTS3 or CCNO. Among the 363 up-regulated genes, 32 are prognostic markers in lung cancer, all of unfavorable prognosis, including LRP8, NUP62CL, FSCN1, PLCD3 or HMGA1 (**Supplementary Table S2**).

**Figure 2:**
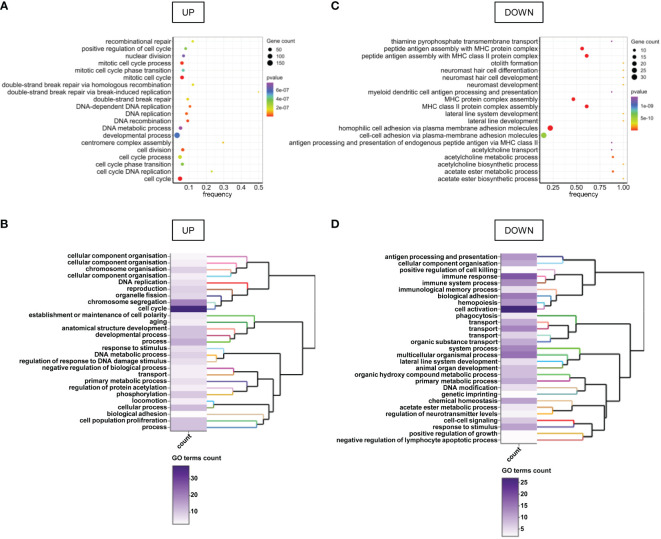
GO terms enriched in genes differentially expressed between FHIT^low^/pHER2^high^ and other tumors. **(A, C)** Bubble charts of the top 20 enriched GO terms for genes up-regulated **(A)** and down-regulated **(C)** in FHIT^low^/pHER2^high^ tumors versus others. Genes are annotated in GO terms with ViSEAGO R package using the Ensembl data base. The horizontal axis represents the frequency of differentially expressed genes in the ontology, i.e. the number of up- or down-regulated genes annotated to the specific ontology divided by the total number of genes in the ontology. The size of the bubble represents the number of genes up- or down-regulated annotated to the ontology and the color depends on the p-value of Fisher’s exact enrichment test. **(B, D)** Heatmap and clustering of the GO clusters for GO terms enriched in genes up- **(B)** or down-regulated **(D)** in FHIT^low^/pHER2^high^ tumors versus others. Clustering was performed on the semantic similarity. The color of the heatmap depends on the number of GO terms in the cluster.

Gene Set Enrichment Analysis (GSEA) revealed that cancer hallmarks related to cell proliferation (E2F targets, G2M checkpoint, MYC targets V1, Mitotic spindle, MTORC1 signaling, P53 pathway, spermatogenesis, MYC targets V2, estrogen response late, PI3K AKT MTOR signaling, androgen response), DNA repair, metabolism (reactive oxygen species pathway, glycolysis, cholesterol homeostasis, hypoxia), immune response (interferon-alpha response, interferon-gamma response, allograft rejection, complement) and metastasis (apical junction, epithelial-mesenchymal transition, apical surface, hedgehog signaling, TGF beta signaling) were significantly enriched in FHIT^low^/pHER2^high^ subclass (**Figures 1C, D**; **Supplementary Figure S3**). Functional profiling showed that genes up-regulated in FHIT^low^/pHER2^high^ tumors were mostly enriched in basic processes such as DNA replication and repair or cell division (**Figures 2A, B**). The signaling pathways they belong to were also related to cell proliferation (**Supplementary Figure S4**). On the other hand, genes down-regulated in FHIT^low^/pHER2^high^ tumors were enriched in terms of transport, cell adhesion, response to diverse stimuli, and immune response essentially related to major histocompatibility complex (MHC) class II (**Figures 2C, D**).

### 3.2 FHIT^low^/pHER2^high^ tumors are of poor prognosis

**Figure 3:**
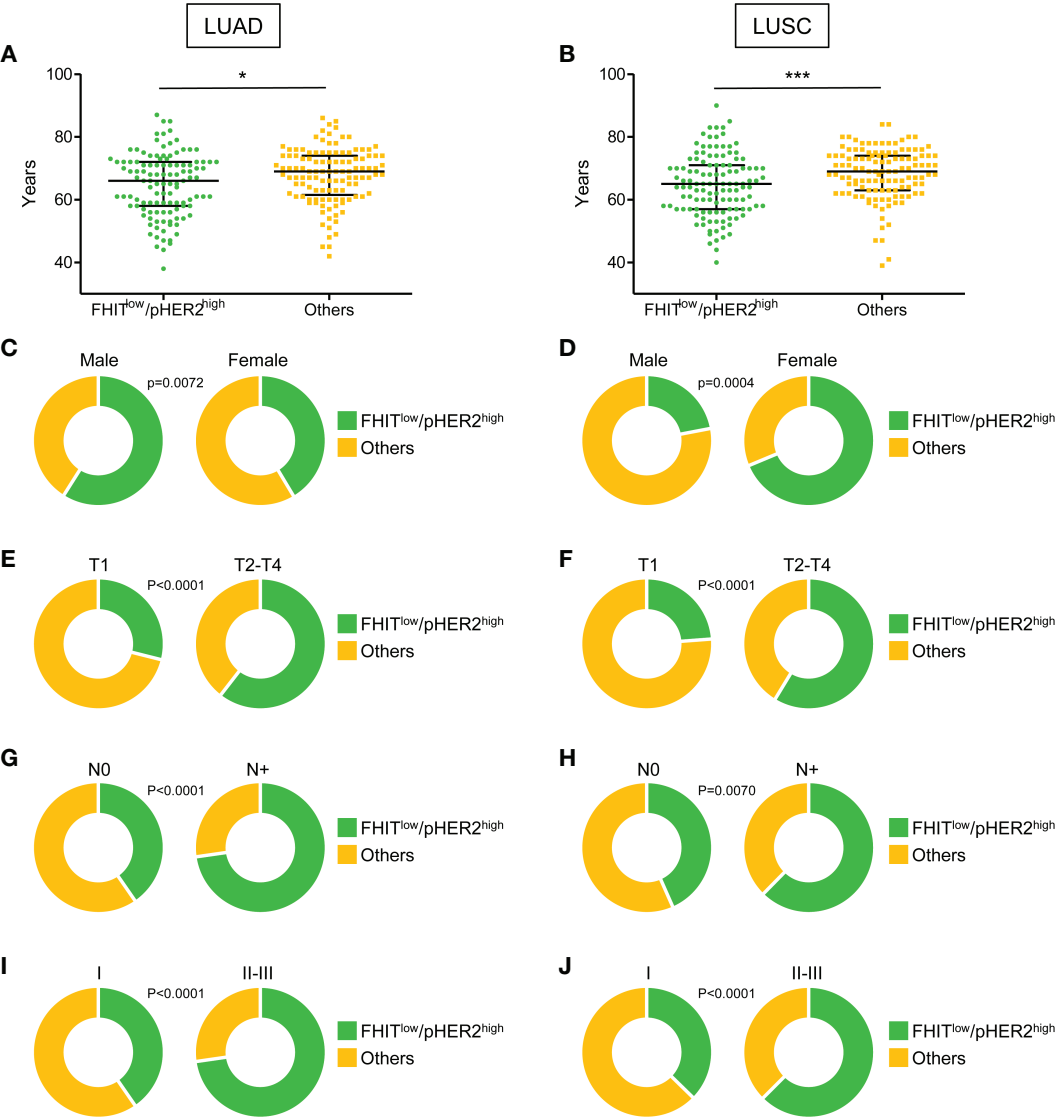
Clinical parameters associated with FHIT^low^/pHER2^high^ signature in Firehose Legacy TCGA-LUAD and TCGA-LUSC cohorts. **(A, B)** Distribution of the age of FHIT^low^/pHER2^high^ (in green) and other patients (in yellow) in LUAD **(A)** and LUSC **(B)** from a two-tailed Mann Whitney test. The median with 1^rst^ and 3^rd^ quartiles are shown in black. *p < 0.05; ***p < 0.001. **(C-J)** Distribution of the sex **(C, D)**, tumor size (T part of the TNM score) **(E, F)**, lymph node involvement (N part of the TNM score) **(G, H)**, and TNM stage **(I, J)** of FHIT^low^/pHER2^high^ (in green) and other patients (in yellow) in LUAD and LUSC, respectively (Fisher’s exact p-values).

Our RNA sequencing analysis suggested a link between FHIT^low^/pHER2^high^ phenotype and a higher aggressiveness in NSCLC. Therefore, the FHIT^low^/pHER2^high^ signature was challenged in LUAD and LUSC cohorts from TCGA (Firehose Legacy) to assess its prognostic and predictive capacity. Tumors were classified as either FHIT^low^/pHER2^high^ or others. In both LUAD and LUSC cohorts, patients with FHIT^low^/pHER2^high^ tumors were significantly younger than others (p=0.0198 and p=0.0004, respectively) (**Figures 3A, B**, **Supplementary Table S3**). Surprisingly, the FHIT^low^/pHER2^high^ phenotype was more frequent in men in LUAD cohort and more frequent in women in LUSC cohort (p=0.0072 and p<0.0001, respectively) (**Figures 3C, D**, **Supplementary Table S3**). Interestingly, FHIT^low^/pHER2^high^ tumors had significantly higher size (p<0.0001 and p<0.0001, respectively) (**Figures 3E, F**, **Supplementary Table S3**), N status (p<0.0001 and p=0.0070, respectively) (**Figures 3G, H**, **Supplementary Table S3**) and TNM stage (p<0.0001 and p<0.0001, respectively) (**Figures 3I, J**, **Supplementary Table S3**) than other tumors in both LUAD and LUSC cohorts.

**Figure 4:**
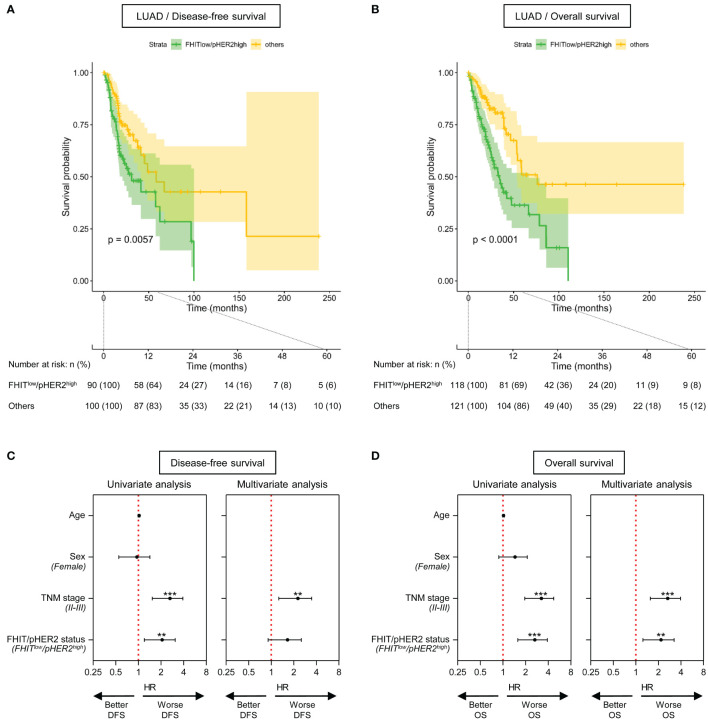
The FHIT^low^/pHER2^high^ signature predicts a poor outcome in Firehose Legacy TCGA-LUAD cohort. **(A, B)** Kaplan-Meier survival curves for disease-free survival **(A)** and overall survival **(B)** by FHIT/pHER2 status. FHIT^low^/pHER2^high^ patients are in green and others in yellow. Each cross is a censored event. Confidence intervals for the curves are colored. Numbers at risk are shown by FHIT/pHER2 status every 12 months for 5 years. Log-rank test p-value for the difference between the curves is written. **(C, D)** Significant variates for disease-free (DFS) **(C)** and overall (OS) **(D)** survival. Female, stages II-III and FHIT^low^/pHER2^high^ were compared to their respective reference Male, stage I, and Others. Hazard risk (HR) and it’s 95% confidence interval **(CI)** are plotted for all tested variates (Wald statistic p-value). The red vertical dashed line corresponds to HR of 1. **p < 0.01; ***p < 0.001.

In LUAD cohort, we also observed that patients with FHIT^low^/pHER2^high^ tumors had significantly shorter disease-free survival (DFS) (p=0.0067) (**Figure 4A**) and overall survival (OS) (p<0.0001) (**Figure 4B**). Univariate analysis revealed that TNM stages II-III and the FHIT^low^/pHER2^high^ phenotype were significantly associated with a worse DFS (HR=2.439 [1.527-3.896], p=0.00019, and HR=1.922 [1.2-3.079], p=0.00654, respectively) (**Figure 4C**, left) and OS (HR=3.036 [1.95-4.728], p<0.0001, and HR=2.474 [1.574-3.887], p<0.0001, respectively) (**Figure 4D**, left). Taking into account both TNM stage and FHIT/pHER2 status effects in a multivariate Cox model, TNM stages II-III were an independent factor for a higher risk of worse DFS (HR=2.0883 [1.26-3.461], p=0.00428) (**Figure 4C**, right) and OS (HR=2.4779 [1.55596-3.0371], p=0.000122) (**Figure 4D**, right), whereas the FHIT^low^/pHER2^high^ phenotype was able to independently predict worse OS (HR=2.007 [1.244-3.239], p=0.004297) but not DFS (**Figures 4C, D**, right). Neither clinical parameters, nor the FHIT/pHER2 status, were found to be associated with DFS and OS in LUSC cohort (data not shown).

### 3.3 FHIT^low^/pHER2^high^ tumors are refractory to ICI

**Figure 5:**
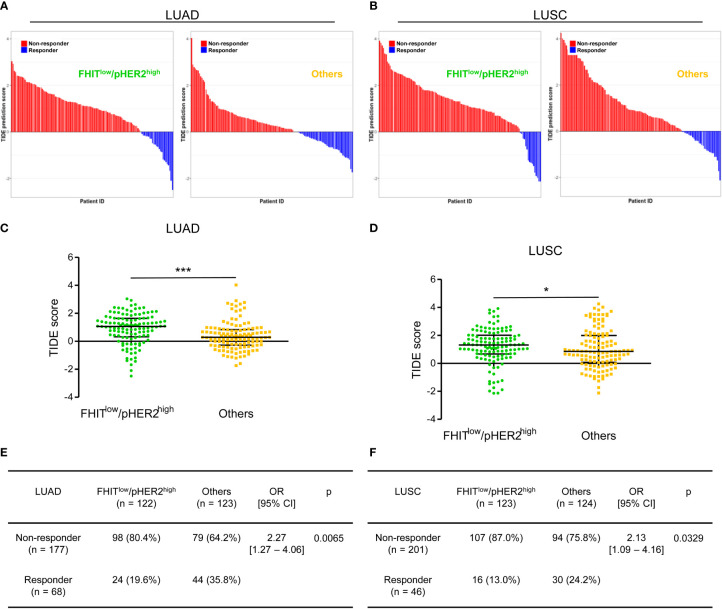
The FHIT^low^/pHER2^high^ signature predicts a poor response to immune checkpoint inhibitors in Firehose Legacy TCGA-LUAD and TCGA-LUSC cohorts. **(A, B)** Waterfall plots of TIDE (Tumor Immune Dysfunction and Exclusion) prediction score across all LUAD **(A)** and LUSC **(B)** tumors. Tumors were separated according to their FHIT/pHER2 status. Blue indicates a tumor that is predicted to respond to therapy. Red indicates a non-responder. In each category, tumors were sorted in descending order according to their TIDE prediction score. **(C, D)** Comparison of TIDE prediction scores by Mann-Whitney test between FHIT^low^/pHER2^high^ tumors and others in LUAD **(C)** and LUSC **(D)** cohorts. *p < 0.05; ***p < 0.001. **(E, F)** Contingency table and Fisher’s exact p-value to estimate the significance of the association between FHIT/pHER2 status and response to immunotherapies in LUAD **(E)** and LUSC **(F)** cohorts.

Since our data suggested an immune escape of FHIT^low^/pHER2^high^ tumors, we also tested the responsiveness of FHIT^low^/pHER2^high^ tumors to immunotherapy in the same LUAD and LUSC TCGA-cohorts. We took advantage of the TIDE (Tumor Immune Dysfunction and Exclusion) computational method. This method that models both induction of T cell dysfunction and prevention of T cell infiltration in tumors was previously demonstrated to predict response to ICI more accurately than other biomarkers (PD-L1 and tumor burden) (28). FHIT^low^/pHER2^high^ tumors were predicted to be less responsive to ICI (**Figures 5A, B**). The FHIT^low^/pHER2^high^ tumors had significantly higher TIDE scores than others in LUAD cohort (p<0.0001) (**Figure 5C**) and LUSC cohort (p=0.0462) (**Figure 5D**). Only 19.6% and 13.0% of FHIT^low^/pHER2^high^ tumors were considered as responder versus respectively 35.8% and 24.2% of other tumors (p=0.0065 and p=0.0329) in LUAD (**Figure 5E**) and LUSC (**Figure 5F**) cohorts.

## 4 Discussion

The analysis of the transcriptomic signature of FHIT^low^/pHER2^high^ NSCLC tumor cells highlighted a distinct NSCLC molecular subclass with potential clinical relevance. First, FHIT^low^/pHER2^high^ NSCLC exhibited higher proliferation and high invasion/metastasis features. This is in agreement with our previous findings showing that growth and invasion induced by FHIT loss are HER2-dependent in lung tumor cells (7). This is also in agreement with their individual role already described in these processes. The tumor suppressor FHIT is well known to control cell proliferation and apoptosis (8, 33). In addition, FHIT impedes tumor invasion and metastasis through its ability to suppress epithelial-mesenchymal transition (EMT) in lung cancer cells (34–36). HER2, as a growth factor orchestrating MAPK/ERK and PI3K/AKT signaling pathways, is a driving factor in the development and progression of lung cancer (5). This profile was also associated with the deregulation of metabolic processes such as glycolysis and ROS production. Metabolic reprogramming is an important mechanism employed by cancer cells to sustain tumor initiation, progression, and metastasis (37). Interestingly, it was shown that FHIT could be located in mitochondria and modulate ROS generation (38). Altogether, these data suggest a particularly aggressive profile for FHIT^low^/pHER2^high^ tumors. This was confirmed by testing our signature in TCGA LUAD and LUSC cohorts. We observed that the FHIT^low^/pHER2^high^ signature was associated with the higher size of tumors, lymph node involvement, and advanced TNM stage in LUSC and LUAD cohorts, with shorter DFS and OS in LUAD. Thus, the FHIT^low^/pHER2^high^ signature could be a relevant biological prognostic surrogate, helping to determine patients eligible for adjuvant therapies in early stages or worse prognosis in later stages.

After observing a specific worse prognosis for this molecular subclass, we investigated how this condition could impact ICI sensitivity. Using TIDE prediction model, we found that FHIT^low^/pHER2^high^ tumors were primarily poor responders to ICI. This is in line with our functional analysis data showing deregulation of immune response especially a down-regulation of MHC class II in this type of tumor. Indeed, tumor-specific (ts) MHC-II is associated with a better prognosis and a better response to ICI (39). Interestingly, it was previously demonstrated that MHC class I expression is positively regulated by FHIT on mouse tumor cells but no link between FHIT and MHC class II has been yet established (40, 41). Moreover, EMT, a hallmark of FHIT^low^/pHER2^high^ tumors, is involved in immunotherapy resistance (42). Furthermore, our results suggest that phenotypic plasticity could lead to the same consequences as genetic alterations since the results of retrospective studies did not argue in favor of the use of ICI as a therapeutic strategy in NSCLC patients carrying HER2 mutations (5). ICI poor sensitivity for FHIT^low^/pHER2^high^ tumors seems comparable to disappointing immune response in NSCLC with oncogenic driver alterations (43, 44).

In addition, it is noteworthy that several different prognostic or predictive signatures have already been published for NSCLC. This new signature, common to adenocarcinomas and squamous cell carcinomas, has the advantage to predict both response to ICI and response to anti-HER2 targeted therapy of NSCLC.

A limitation of this study is the test of the signature on TCGA cohorts without clinical data on ICI activity. It would be interesting to evaluate FHIT/pHER2 status in clinical cohorts treated with ICI or anti-HER2 targeted therapy. NSCLC were non-metastatic cases and the extrapolation of our data to metastatic NSCLC remains to be demonstrated. Of notes, the management of early-stage resected NSCLC currently benefits from substantive progress, with growing interest for TKI and ICI in a peri-operative context (45, 46).

In conclusion, we showed that the transcriptomic signature associated with the FHIT^low^/pHER2^high^ molecular subclass was a new relevant condition associated with poor prognosis and low sensitivity to immunotherapy. These data suggest the need for further exploration of the FHIT^low^/pHER2^high^ status in NSCLC, both in the late and early stages to better select patients eligible for ICI. They also reinforce the concept that targeting HER2 could be of therapeutic value for NSCLC patients with this subtype of tumors. The relevance of this new subclass should be investigated in prospective clinical trials.

## Data availability statement

The datasets presented in this study can be found in online repositories. The names of the repository/repositories and accession number(s) can be found below: https://www.ncbi.nlm.nih.gov/geo/ under the accession number GSE208544.

## Ethics statement

The studies involving human participants were conducted in accordance with the ethical guideline of the Declaration of Helsinki. Access to patient data was approved by the French Commission Nationale de l'Informatique et des Libertés. The patients/participants provided their written informed consent to participate in this study.

## Author contributions

BN-R and MP designed the study. TP, EL, AG and BN-R conducted the experiments. AB performed all bioinformatic analyses. AB, JA, and BN-R analyzed the data. VeD, VaD, GaD, and MP contributed to experiment analysis and interpretation of the results. GoD, AD and GaD provided clinical resources. AB and BN-R wrote the manuscript. All authors contributed to the article and approved the submitted version.

## Funding

This work was supported by grants from La Ligue Contre le Cancer (Committees of Ardennes, Doubs-Montbéliard, Marne, and Meuse) and Lions Club of Soissons, Villers-Cotterets and Crépy-en-Valois. AB was supported by the Fondation pour la Recherche Médicale. TP was supported by the University of Reims Champagne-Ardenne. EL was supported by the Région Grand Est and INSERM.

## Acknowledgments

The results shown here are in part based upon data generated by the TCGA Research Network: https://www.cancer.gov/tcga.

## Conflict of interest

The authors declare that the research was conducted in the absence of any commercial or financial relationships that could be construed as a potential conflict of interest.

## Publisher’s note

All claims expressed in this article are solely those of the authors and do not necessarily represent those of their affiliated organizations, or those of the publisher, the editors and the reviewers. Any product that may be evaluated in this article, or claim that may be made by its manufacturer, is not guaranteed or endorsed by the publisher.

## Abbreviations

ADC, adenocarcinoma; Ap3A, diadenosine triphosphate; DFS, disease-free survival; EMT, epithelial-mesenchymal transition; FDR, false discovery rate; FHIT, fragile histidine triad; ICI, immune checkpoint inhibitor; LUAD, lung adenocarcinoma; LUSC, lung squamous cell carcinoma; GO, gene ontology; GSEA, gene set enrichment analysis; HR, hazard risk; MHC, major histocompatibility complex; NSCLC, non-small cell lung cancer; OS, overall survival; PD-L1, programmed death-ligand 1; SCC, squamous cell carcinoma; TCGA, the cancer genome atlas; TIDE, tumor immune dysfunction and exclusion; TKI, tyrosine kinase inhibitor; TNM, tumor node metastasis.
