# Supplementary material for: Transcriptomic FHITlow/pHER2high signature as a predictive factor of outcome and immunotherapy response in non-small cell lung cancer
Source: Front Immunol. 2022 Dec 5;13:1058531. doi: 10.3389/fimmu.2022.1058531 (PMC9760670; doi:10.3389/fimmu.2022.1058531)
Supplement: Supplementary file 1 [file DataSheet_1.pdf]

Supplementary Material

Supplementary figure S1: Selection of the samples for RNA-sequencing analysis from a NSCLC cohort.

Supplementary figure S2: Heatmap of all expressed mRNAs.

Supplementary figure S3: Enrichment plots from GSEA analysis of significantly enriched cancer's hallmarks from Msig database in $FHIT^{low}/pHER2^{high}$ tumors.

Supplementary figure S4: Reactome pathways enriched in genes differentially expressed between $FHIT^{low}/pHER2^{high}$ and other tumors.

Supplementary table S1: Description of the population studied by RNA sequencing.

Supplementary table S2: Genes significantly differentially expressed.

Supplementary table S3: Factors associated with $FHIT^{low}/pHER2^{high}$ signature in NSCLC.

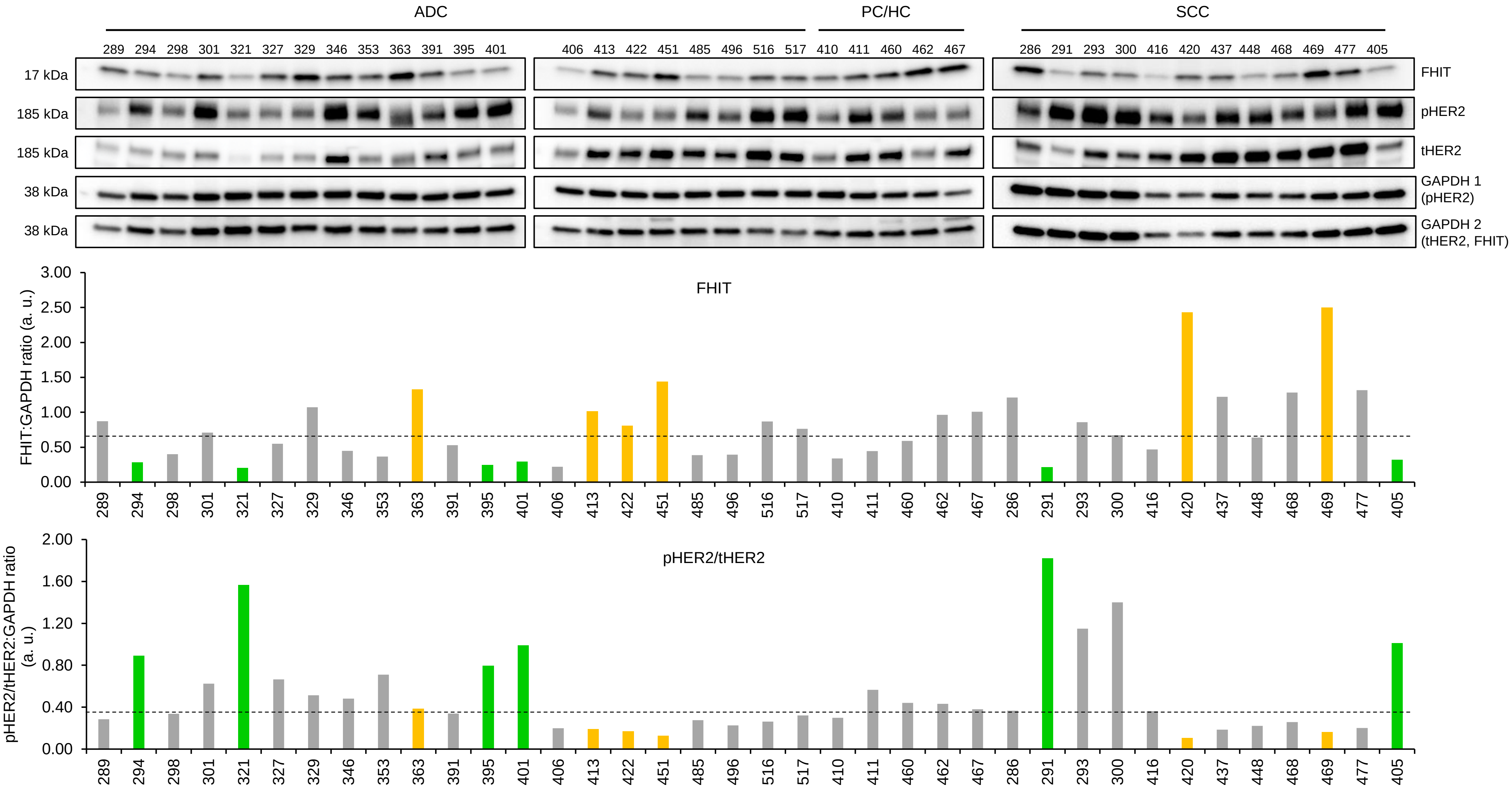

Supplementary figure S1: Selection of the samples for RNA-sequencing analysis from a NSCLC cohort. Western blot analysis of FHIT, pHER2 and tHER2 levels in a series of NSCLC primary cultures. Levels of GAPDH served as loading controls. Samples were selected depending on their relative expression of FHIT and pHER2 revealed by western blot quantification. Horizontal dashed lines represent respectively the median of FHIT level and of pHER2/tHER2 ratio. Selected FHIT^{low}/pHER2^{high} samples are shown in green and others in yellow. Abbreviations: ADC, adenocarcinoma; PC, pleomorphic carcinoma; HC: hybrid carcinoma; SCC, squamous cell carcinoma.

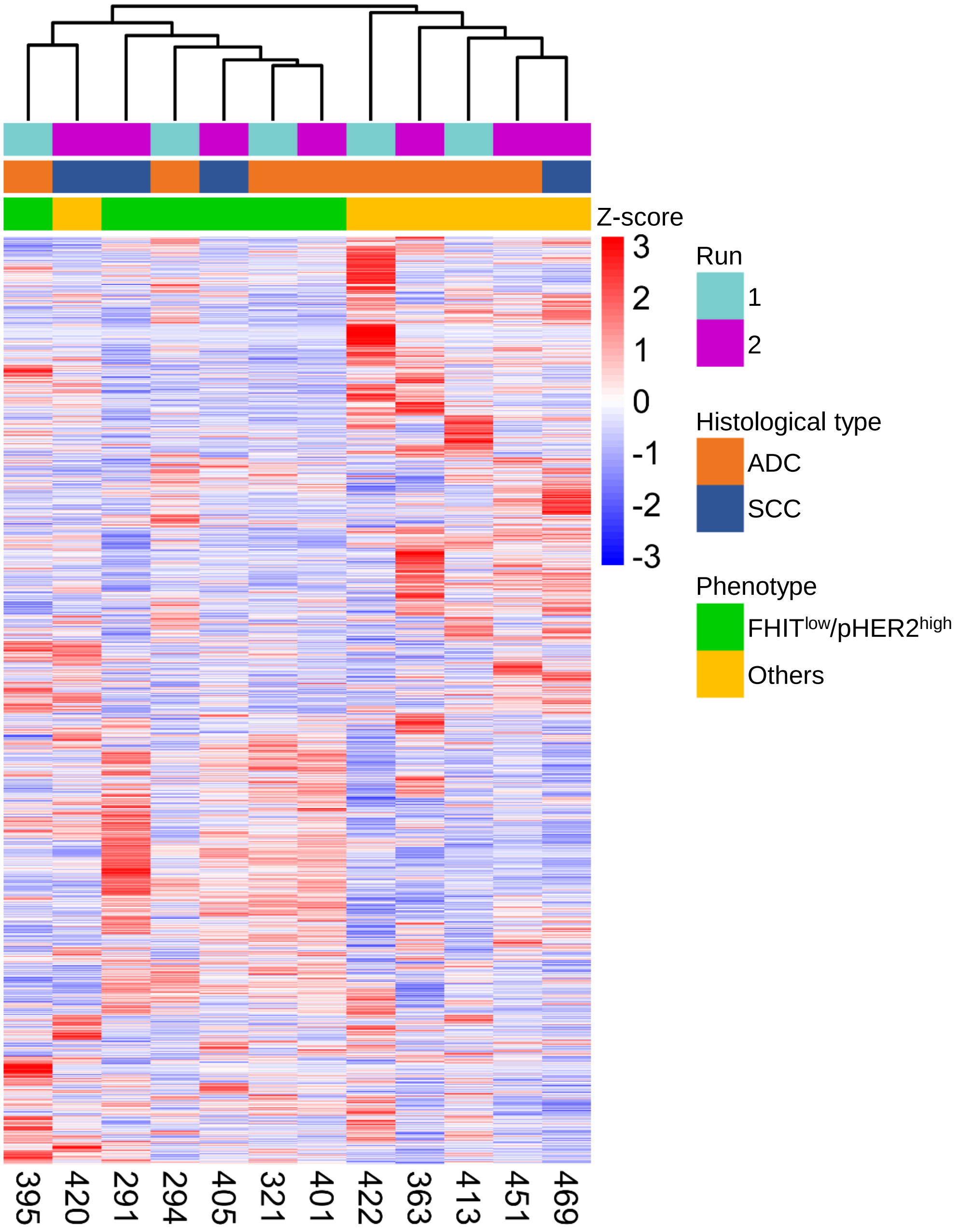

Supplementary figure S2: Heatmap of all expressed mRNAs. Scaled rlog transformed values are plotted in FHIT^{low}/pHER2^{high} (green) against other (yellow) tumor samples. Each column represents a tumor, whereas each line represents a gene. Expression values by row (by gene) are centered so that the color reflects the amount by which each gene deviates in a specific sample from the gene's average across all samples. The patients are clustered in an unsupervised hierarchical way. The top of the graph shows the sample's membership. FHIT^{low}/pHER2^{high} tumors are green and others are yellow. Data were sequenced in two times plotted respectively in light blue and purple. Histological type of the tumor is separated in adenocarcinomas (ADC) in orange and squamous cell carcinomas (SCC) in dark blue.

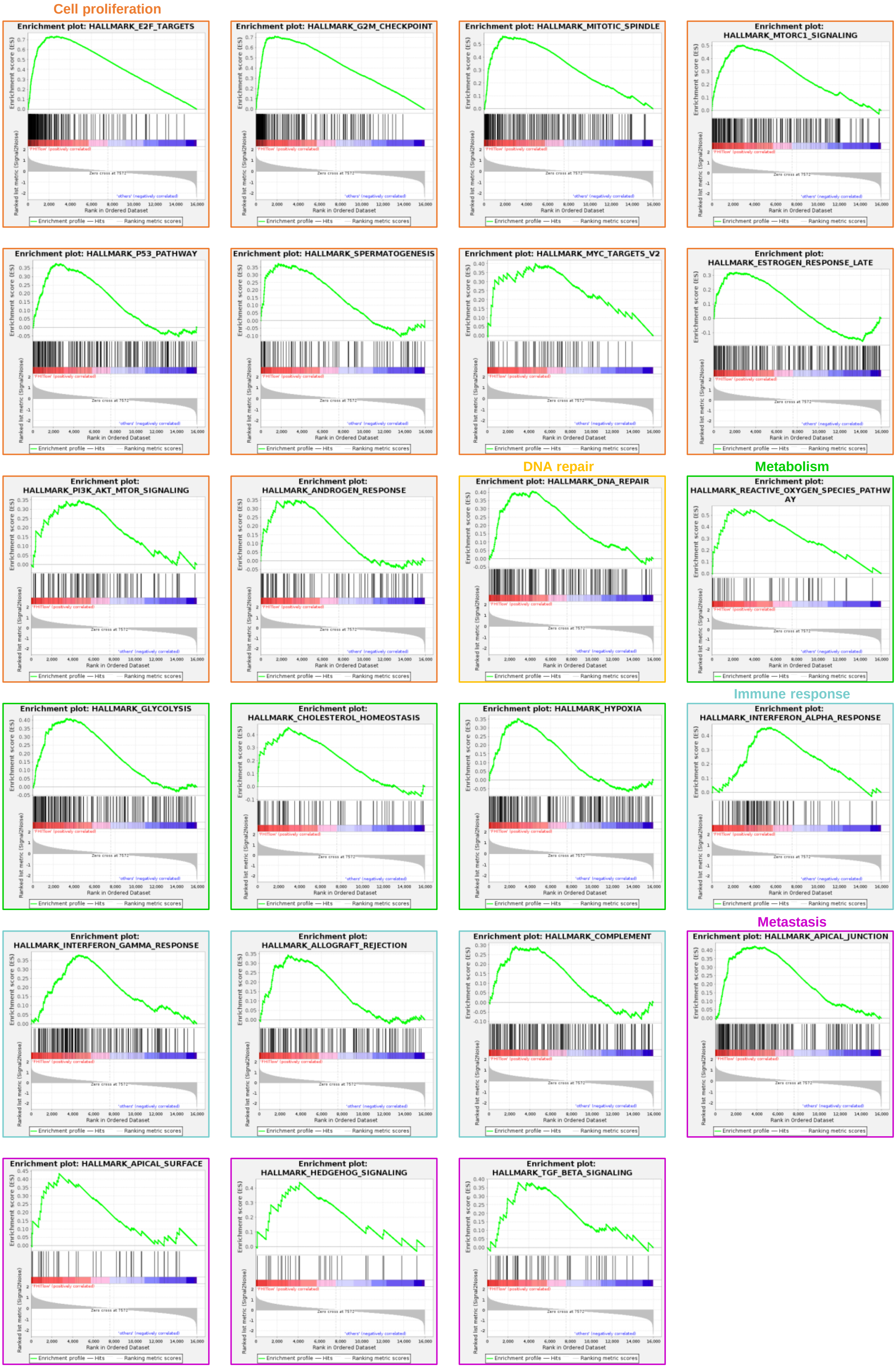

Supplementary figure S3: Enrichment plots from GSEA analysis of significantly enriched cancer's hallmarks from Msig database in FHIT^{low}/pHER2^{high} tumors. Hallmarks are grouped and colored depending on the cancer process they belong to.

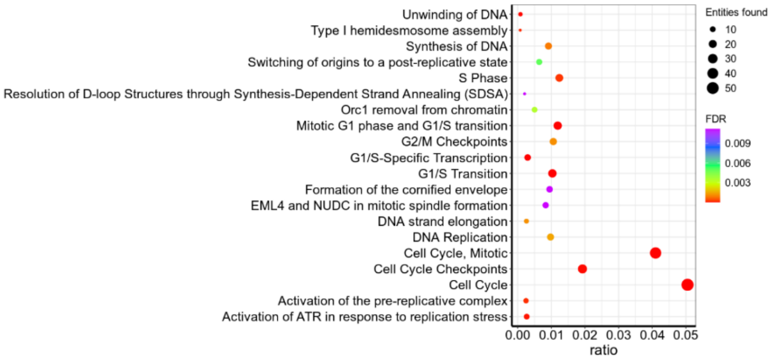

Supplementary figure S4: Reactome pathways enriched in genes differentially expressed between FHIT^{low}/pHER2^{high} and other tumors. Bubble chart of the 20 most enriched Reactome pathways. Reactome pathways are enriched in genes up-regulated in FHIT^{low}/pHER2^{high} tumors versus others. The horizontal axis represents the p-value of over-representation (hypergeometric distribution) test as calculated by the Reactome analysis. The size of dots indicates the number of the submitted entities found in each pathway. The color of each dot represents the false discovery rate (FDR) using the Benjamani-Hochberg method of over-representation test.

Supplementary table S1: Description of the population studied by RNA sequencing

		$FHIT^{low}/pHER2^{high}$ (n=6)	Others (n=6)	*p*
Age (years)		62.5	67.5	NS[(a)]
Sex	Female	3	3	NS[(b)]
	Male	3	3	
Histological type	ADC	4	4	NS[(b)]
	SCC	2	2	
Differentiation status	Well	0	3	NS[(b)]
	Mild	2	1	
	Poor	4	2	
TNM stage	I	2	0	NS[(b)]
	II	1	5	
	III	2	1	
	Unknown	1	0	
Tucatinib IC50 (nM)		100.04	649.45	0.0022[(a)]

nM: nano Mole; [a] p-value from the Mann-Whitney test; [b] p-value from the Fisher's exact test

Supplementary table S2: Genes significantly differentially expressed
Unfavorable prognostic markers in lung cancer are indicated in red
Favorable prognostic markers in lung cancer are indicated in blue

	baseMean	log2FoldChange	lfcSE	stat	pvalue	padj
ABCC6	164.598078	-2.759781443	0.73958821	-3.73151088	0.00019033	0.00473061
ABCG1	453.512234	-1.410298623	0.41704151	-3.38167446	0.00072045	0.01165606
ABLIM3	852.286931	1.995540471	0.55498118	3.59569032	0.00032353	0.00681748
AC011747.4	10.8764966	-2.991964403	0.98168609	-3.04778118	0.00230538	0.02503788
AC017060.1	29.4800596	-2.578808696	0.64795767	-3.97990304	6.89E-05	0.00241364
AC074289.1	9.08672208	1.956275816	0.69496069	2.81494455	0.00487857	0.04041756
ACAP1	17.2678801	1.549402775	0.53365984	2.90335276	0.00369191	0.03388033
ACCS	115.2266	-1.279419281	0.33764907	-3.78919834	0.00015113	0.00408272
ACE	56.481075	1.365639601	0.48884039	2.79363087	0.00521199	0.04249885
ACKR2	106.771531	-1.491212404	0.47227224	-3.15752714	0.00159113	0.01966024
ACOX2	66.6997382	-2.109573697	0.42821892	-4.92639069	8.38E-07	0.00010476
ACP5	744.342835	1.043044373	0.28671847	3.63786946	0.0002749	0.00608818
ACTBL2	99.4976522	-1.91316669	0.49313116	-3.87963054	0.00010462	0.0032192
ADA	1489.40075	1.713560892	0.49556213	3.4578124	0.00054458	0.0097019
ADAM23	24.7906458	2.566111379	0.91576905	2.80213813	0.00507651	0.0417453
ADAMTS1	6202.76388	1.977120078	0.43203601	4.57628535	4.73E-06	0.00035983
ADAMTS9	113.150999	-2.89564685	0.82687293	-3.50192483	0.00046191	0.00868335
ADARB2	218.371784	-2.44352298	0.77081305	-3.17005919	0.00152408	0.01909353
ADPRHL1	62.5476199	-1.654342569	0.50368132	-3.28450251	0.00102163	0.01460313
ADRA2A	31.0717066	-6.182460133	1.26638294	-4.8819831	1.05E-06	0.00012247
ADRB1	78.910685	-1.610005302	0.57962617	-2.77766152	0.00547516	0.04395149
AGAP2-AS1	215.270093	1.318256615	0.34426968	3.82913945	0.00012859	0.00366401
AGR2	8497.06821	-1.379512251	0.4437444	-3.10879925	0.00187849	0.02181869
AGR3	51.7198566	-2.81193976	1.00140728	-2.80798813	0.00498521	0.0411253
AKR1B10	2858.74999	1.780489894	0.5528359	3.22064811	0.00127901	0.01698148
AKT3	421.799666	1.221979225	0.41751437	2.92679561	0.00342474	0.03230795
ALDH3B1	1760.57941	-1.368693497	0.4194458	-3.26309976	0.00110201	0.01537561
ALDH3B2	1508.81922	-1.560042408	0.54252745	-2.8755087	0.00403377	0.03558938
ALDH4A1	2661.6646	1.062529467	0.3415124	3.11124706	0.00186299	0.02167525
ALDOC	1643.92105	1.207496353	0.41774884	2.89048403	0.00384649	0.03464299
ALPL	901.268001	-1.433042984	0.51887246	-2.7618405	0.00574765	0.04527191
AMIGO1	46.0540671	-1.28484731	0.4588658	-2.80005026	0.00510947	0.04186091
AMN	229.427885	-2.729743208	0.48840637	-5.58908192	2.28E-08	6.81E-06
AMOT	335.652506	-1.406678408	0.46330485	-3.03618318	0.00239594	0.02573306
AMY2B	25.9722867	-1.690927817	0.46279121	-3.65375959	0.00025843	0.00580024
ANKRD18A	63.9667157	1.134589324	0.25371195	4.47195862	7.75E-06	0.0005161
ANKRD36C	59.0706753	-1.5206031	0.40133615	-3.78885161	0.00015135	0.00408272
ANLN	6152.57916	1.766023317	0.60920217	2.89891171	0.0037446	0.03420203
ANO1	625.391693	1.979114079	0.45425801	4.3568061	1.32E-05	0.00076947
ANPEP	549.356375	-3.099713929	0.59484311	-5.21097728	1.88E-07	3.54E-05
AP3B2	29.8733995	-1.974886871	0.64117377	-3.08011177	0.00206923	0.02328859
APOBEC3A	24.5293318	-2.167962373	0.69098497	-3.1374957	0.00170398	0.02042271
APOBR	33.4991153	-2.236753627	0.35848803	-6.23940945	4.39E-10	4.01E-07
ARHGAP11A	1773.59697	1.535753477	0.50783015	3.0241479	0.00249335	0.02630564
ARHGAP11B	63.7338403	1.684186882	0.41829635	4.02630068	5.67E-05	0.00210229
ARHGAP19	603.078165	1.025575857	0.2139026	4.79459278	1.63E-06	0.00016742
ARHGAP26	662.596994	-1.636005054	0.50808573	-3.21993898	0.00128218	0.01698148
ARHGAP28	125.797245	-2.120030885	0.76950499	-2.75505801	0.00586817	0.04585197
ARHGAP4	212.986861	-2.466335415	0.63963205	-3.85586592	0.00011532	0.00341318
ARHGAP44	674.14352	-1.327917881	0.32128209	-4.13318368	3.58E-05	0.00151723
ARHGEF38	143.867721	-3.277926988	0.53937294	-6.07729227	1.22E-09	8.24E-07

ARNTL2	4500.06201	1.074220853	0.22324304	4.81188963	1.50E-06	0.00016053
ARRB1	556.983363	-3.019485058	0.51729839	-5.83702777	5.31E-09	2.42E-06
ARSI	414.856144	1.773512579	0.59920029	2.95979924	0.0030784	0.03006476
ASAH1	7667.86168	-1.122621926	0.1701925	-6.59618911	4.22E-11	8.18E-08
ASCL3	53.1847582	-7.871473725	2.13348999	-3.68948238	0.00022471	0.00528838
ASMTL-AS1	39.3366224	-1.226752909	0.35823999	-3.42438854	0.00061618	0.01058712
ATAD3C	65.498146	-1.345306864	0.44662582	-3.0121565	0.00259399	0.02689182
ATAD5	204.664675	1.234066081	0.37215491	3.31600108	0.00091315	0.0137097
ATG9B	20.4820028	-1.930385807	0.55040704	-3.5071968	0.00045285	0.00858596
ATP12A	8772.26945	-2.290697728	0.78629451	-2.91328209	0.00357651	0.03325429
ATP13A5	26.1389882	-4.108766602	0.88202784	-4.65831849	3.19E-06	0.00027468
ATP1B1	19126.1042	-1.121733531	0.31161723	-3.59971595	0.00031856	0.00676798
ATP2A3	210.984517	-3.272421129	0.88359263	-3.70354056	0.00021261	0.00510431
ATP6V0A4	39.8997107	-2.384322564	0.85878479	-2.77639124	0.0054966	0.0440552
ATP6V0E2	452.511136	-1.01934693	0.23735283	-4.29464833	1.75E-05	0.00093103
ATP6V0E2-AS1	18.9038506	-1.8257208	0.45151703	-4.04352585	5.27E-05	0.00199658
ATP6V1C2	281.111617	-3.294172887	1.09282994	-3.01435089	0.0025753	0.02679795
ATP8B3	97.8831554	1.521619471	0.38101853	3.99355771	6.51E-05	0.0023153
AWAT2	8.46127684	-2.522331845	0.82755142	-3.04794576	0.00230412	0.02503788
AXIN2	343.284945	-1.70349851	0.59669473	-2.85489118	0.00430516	0.03710263
AZGP1	1836.15589	-3.891648292	0.65446196	-5.94633234	2.74E-09	1.35E-06
B4GALNT1	31.4247123	2.799993478	0.94023089	2.977985	0.0029015	0.02905061
B4GALNT3	437.018322	-1.712571195	0.48822399	-3.50775718	0.0004519	0.00857838
BAALC	23.4379124	-1.495882336	0.51682986	-2.89434191	0.00379954	0.03439566
BARX2	1149.76379	1.382645783	0.43344341	3.18991073	0.00142317	0.01812143
BATF	81.357887	-2.026531194	0.50949232	-3.97755	6.96E-05	0.00241583
BCL2L12	588.166128	1.087731627	0.23898023	4.55155491	5.33E-06	0.00038058
BCL2L15	204.342912	-2.349820694	0.63108861	-3.7234402	0.00019653	0.00482239
BEND5	49.4281535	-1.167325681	0.40947745	-2.85076918	0.00436136	0.0374117
BEND7	390.068722	-1.016436887	0.17968359	-5.65681539	1.54E-08	4.98E-06
BEX2	138.236783	-1.100716419	0.40360542	-2.72720921	0.00638725	0.04839598
BHLHE41	581.091243	1.157446929	0.34220388	3.38233144	0.00071873	0.01165096
BLACAT1	117.71035	1.714859692	0.43792244	3.91589823	9.01E-05	0.00287876
BMF	374.525636	-1.154514671	0.37443269	-3.08337043	0.0020467	0.02318882
BMP4	194.749146	-1.356113815	0.49696483	-2.72879232	0.00635667	0.04831495
BMP6	9.28854088	-1.854120442	0.67456817	-2.74860353	0.00598497	0.04641047
BMS1P5	21.7446131	-1.049787843	0.33849648	-3.10132574	0.00192656	0.02221491
BNC1	4686.2449	1.026530077	0.28842335	3.55910872	0.00037212	0.00755385
BPIFA1	10448.5296	-5.107944152	1.18209761	-4.32108492	1.55E-05	0.00086
BPIFA2	120.750584	-2.85924778	1.01555852	-2.81544365	0.00487099	0.04039799
BPIFB1	12996.1678	-5.229148106	1.01973739	-5.12793605	2.93E-07	4.99E-05
BRCA1	583.366636	1.4894699	0.39627938	3.75863588	0.00017084	0.00444562
BRIP1	313.8551	1.44520177	0.31347952	4.61019521	4.02E-06	0.00031832
BSPRY	509.495743	-1.510405969	0.48105713	-3.13976424	0.00169084	0.02033659
BTBD11	1593.45294	1.304081126	0.30872283	4.22411621	2.40E-05	0.0011412
BZRAP1	47.0294901	-1.421973564	0.4293995	-3.31153985	0.00092784	0.01382491
C12orf74	36.1603579	-1.632344798	0.55750494	-2.92794681	0.00341208	0.03224742
C12orf75	679.214191	1.64914436	0.28995229	5.68764037	1.29E-08	4.45E-06
C15orf62	249.621725	-1.520511429	0.55407111	-2.74425323	0.00606487	0.04677281
C16orf89	117.814911	-4.174538487	0.78966501	-5.28646759	1.25E-07	2.69E-05
C17orf103	453.188363	-1.04029866	0.27134793	-3.83381831	0.00012617	0.00362363
C18orf54	389.15243	1.528437607	0.35688577	4.28270824	1.85E-05	0.00095768
C18orf56	69.0843463	1.627822894	0.3640994	4.47081999	7.79E-06	0.00051644
C1QL1	30.4614988	2.098189481	0.71125668	2.94997508	0.003178	0.03074706
C1orf112	296.414458	1.044979204	0.33889308	3.0835071	0.00204576	0.02318882

C1orf168	74.0472263	-3.209947078	0.7652353	-4.19471902	2.73E-05	0.00125361
C1orf213	20.9126522	1.778021936	0.42517026	4.18190568	2.89E-05	0.00129575
C2	145.470029	-1.81161727	0.65550543	-2.7636953	0.00571509	0.04517599
C2CD4A	148.237939	-1.549592504	0.39580696	-3.91502088	9.04E-05	0.00287876
C2CD4B	44.4740505	-2.626397905	0.56785671	-4.62510672	3.74E-06	0.00030401
C2orf15	103.985464	-1.917868839	0.32957354	-5.81924402	5.91E-09	2.55E-06
C2orf54	364.0283	-1.681307064	0.57335347	-2.93240933	0.00336343	0.03186527
C3	56958.1841	-1.521308446	0.43350834	-3.50929454	0.0004493	0.00854987
C3orf35	6.93468301	-2.103110851	0.61591796	-3.41459577	0.00063877	0.010843
C3orf67	202.654331	1.76601182	0.30831265	5.72799014	1.02E-08	3.99E-06
C4orf19	696.614481	-1.73068004	0.53746518	-3.22007843	0.00128156	0.01698148
C6orf222	8.96926764	-2.910679612	0.95021292	-3.06318673	0.00218993	0.02419066
C7orf61	9.70694246	1.646989967	0.52108212	3.16071094	0.00157385	0.01954574
C8orf4	3848.8967	-1.887759336	0.66086072	-2.85651617	0.00428318	0.03696598
C8orf47	47.9689434	-2.250239116	0.49293889	-4.56494537	5.00E-06	0.00036792
C8orf88	44.6833982	2.360397467	0.50131625	4.70840011	2.50E-06	0.00022512
C9orf116	45.1089964	-1.10269871	0.24108458	-4.57390802	4.79E-06	0.0003604
CA2	1007.82775	2.449204685	0.57910037	4.22932675	2.34E-05	0.00112197
CA8	54.7176912	-2.640933809	0.79954617	-3.30304104	0.00095642	0.01400678
CABLES1	98.7003363	-1.268843198	0.46574683	-2.72431955	0.00644341	0.04853945
CACNA1A	14.192742	-2.542609968	0.89613441	-2.83730871	0.00454956	0.03851481
CACNA1D	127.138841	-1.379774231	0.50889834	-2.71129641	0.00670207	0.04985246
CACNB4	81.4734635	-1.668857174	0.52688194	-3.16742148	0.00153797	0.01918939
CACNG4	65.8448224	-3.002315766	0.97324332	-3.08485628	0.00203651	0.02315554
CALML3	809.567645	2.528965363	0.74530891	3.39317743	0.00069087	0.01136347
CAMK4	21.7608181	2.941194926	0.904246	3.25264908	0.00114335	0.01571999
CAPN12	203.038926	-1.125976995	0.35521753	-3.16982382	0.00152531	0.01909353
CAPN13	671.802286	-3.491212011	0.56084766	-6.22488463	4.82E-10	4.15E-07
CAPN8	53.0865478	-3.43347465	0.75352687	-4.55653909	5.20E-06	0.00037513
CAPN8-2	93.5962496	-3.164596361	0.80501548	-3.93110002	8.46E-05	0.00276669
CAPN9	20.8058137	-2.037223122	0.7148638	-2.84980597	0.00437459	0.03748372
CAPRIN2	794.819905	1.121414336	0.34198653	3.27911841	0.00104132	0.01480734
CARD10	2048.57753	1.105108588	0.2906001	3.80284993	0.00014304	0.00393337
CASC15	43.1408159	-2.7049947	0.84229096	-3.21147302	0.00132056	0.01728323
CATSPERB	47.6924632	-3.451674115	0.60284049	-5.72568394	1.03E-08	3.99E-06
CAV1	26297.6607	1.191705442	0.31296229	3.80782443	0.00014019	0.00387572
CBR3	221.718695	1.774588685	0.44206033	4.01435855	5.96E-05	0.00215994
CBX7	234.724687	-1.073813407	0.3348797	-3.20656466	0.0013433	0.01755119
CCAT1	187.870074	1.632538291	0.48020008	3.39970436	0.00067459	0.01121348
CCDC109B	412.792934	1.401280789	0.24806872	5.64876057	1.62E-08	5.12E-06
CCDC152	31.612511	1.82353327	0.57312553	3.1817345	0.00146396	0.01848905
CCDC159	191.073365	-1.002742223	0.28162191	-3.5605974	0.00037001	0.00753086
CCDC160	53.2416133	-1.071761464	0.3275065	-3.27248916	0.00106605	0.01505771
CCDC163P	59.8619185	-1.076148466	0.33990833	-3.16599611	0.00154553	0.01926817
CCDC171	55.1236271	-1.076938068	0.37497467	-2.87202883	0.00407846	0.03577647
CCDC40	32.1893946	-2.382613016	0.61390419	-3.88108281	0.00010399	0.0032064
CCDC87	13.461116	-1.609683208	0.41993622	-3.83316115	0.00012651	0.00362661
CCL22	96.7163547	-2.197748659	0.76538913	-2.87141348	0.00408641	0.03581115
CCNA1	1526.65381	1.467901862	0.4491906	3.26788194	0.00108356	0.01520802
CCNF	962.779054	1.452359951	0.48308906	3.00640205	0.00264359	0.02720602
CCNO	591.474949	-1.53208957	0.33266915	-4.60544533	4.12E-06	0.00032239
CCRL2	12.4442176	-2.276942867	0.7006113	-3.2499374	0.0011543	0.01582856
CCSER1	66.2384606	-1.690098816	0.37050045	-4.56166464	5.07E-06	0.00037126
CCT6B	63.5116528	-1.378588157	0.27244228	-5.06011084	4.19E-07	6.29E-05
CD109	11807.6686	1.116932328	0.26495288	4.21558862	2.49E-05	0.00117796

CD55	2523.89495	-1.401469067	0.43521708	-3.22016098	0.00128119	0.01698148
CD7	235.666681	-5.738203508	1.30224586	-4.40639028	1.05E-05	0.00063315
CD74	9508.42643	-1.322483583	0.44456631	-2.97477241	0.00293206	0.0292245
CDC25A	354.057724	1.295531711	0.33685627	3.84594808	0.00012009	0.0034877
CDC45	238.488651	1.613503519	0.50408029	3.20088594	0.00137006	0.01777873
CDC6	496.555316	1.600629613	0.34456206	4.64540293	3.39E-06	0.00028188
CDCA7	647.623291	2.227123818	0.42804354	5.20303102	1.96E-07	3.62E-05
CDH13	2132.56008	1.86831059	0.41860942	4.46313554	8.08E-06	0.00053078
CDHR1	329.021638	1.753473924	0.28323771	6.1908208	5.99E-10	4.64E-07
CDKL1	54.5637539	1.167843436	0.42550173	2.74462677	0.00605798	0.04677232
CDKN1C	648.306514	-1.082605973	0.3279627	-3.30100339	0.0009634	0.01406905
CDT1	719.720931	1.715892246	0.45172597	3.79852471	0.00014556	0.00397189
CEACAM6	18382.4937	-1.983284739	0.6788028	-2.92173917	0.00348083	0.03263856
CEACAM7	16.5416032	-2.201391488	0.70353965	-3.12902262	0.00175389	0.02078002
CELF6	7.39658545	-2.855720947	0.67458987	-4.23326981	2.30E-05	0.00111277
CENPE	1690.33826	1.58209408	0.57803706	2.73701148	0.00620001	0.04739083
CENPH	523.555957	1.315669371	0.34793184	3.78139973	0.00015595	0.00414146
CENPI	281.681281	1.758234791	0.59591235	2.95049227	0.00317268	0.03071479
CENPK	263.79825	1.535276256	0.49961755	3.07290296	0.00211987	0.02367862
CENPP	96.1931359	1.235369872	0.3451084	3.57965748	0.00034404	0.00713341
CENPU	689.93611	1.235400656	0.35814077	3.44948348	0.00056166	0.00991946
CENPW	542.477967	1.56233007	0.44349619	3.52275874	0.00042708	0.00826914
CEP128	195.307464	1.073091893	0.38365316	2.79703648	0.00515737	0.04218653
CFD	266.040968	-2.347169666	0.69885447	-3.35859579	0.0007834	0.01227104
CFTR	390.906595	-2.858437935	0.72023732	-3.96874453	7.23E-05	0.00246642
CHAF1B	364.084899	1.026195093	0.31424444	3.26559508	0.00109234	0.01528985
CHEK1	677.730962	1.416936245	0.47076133	3.0098824	0.00261349	0.02698575
CHKA	899.9677	-1.468660712	0.32943631	-4.45810213	8.27E-06	0.00053503
CHL1	1097.85343	-2.21586759	0.56122483	-3.94827074	7.87E-05	0.00263679
CHN2	26.822843	-3.123366269	0.8494848	-3.67677713	0.0002362	0.00546924
CHPT1	980.420575	-1.627518169	0.42713897	-3.81027787	0.00013881	0.00384431
CHST3	2370.17124	1.179720949	0.30427629	3.87713733	0.00010569	0.0032395
CHST4	110.060899	-1.721498052	0.63435202	-2.7137898	0.00665184	0.04959775
CHST7	204.889662	1.099646605	0.29014657	3.78996934	0.00015067	0.00408272
CILP	149.167792	-3.453192522	0.82686894	-4.17622717	2.96E-05	0.00132467
CIT	1364.38585	1.601540362	0.590123	2.71390941	0.00664943	0.04959775
CKS1B	799.31436	1.167225648	0.30429992	3.83577375	0.00012517	0.00360159
CKS2	1347.90752	1.189719209	0.40478116	2.93916643	0.00329096	0.03146704
CLCA2	19322.7621	1.354014564	0.29365725	4.61086717	4.01E-06	0.00031832
CLCNKB	36.262099	-6.745376107	1.49438826	-4.51380426	6.37E-06	0.0004389
CLDN23	457.379507	-1.217903024	0.33925121	-3.58997402	0.00033071	0.00691239
CLDN4	23217.434	-1.230240745	0.45140913	-2.72533422	0.00642364	0.04850257
CLGN	363.96793	-2.181442853	0.71550058	-3.04883452	0.00229731	0.02500279
CLIC5	94.6475222	-3.385374502	0.8612922	-3.93057606	8.47E-05	0.00276689
CLIC6	2064.19155	-2.989739314	0.50422145	-5.92941716	3.04E-09	1.43E-06
CLYBL	222.757762	-1.13657341	0.31652603	-3.59077392	0.0003297	0.00690816
CNTLN	291.736425	1.176194902	0.35988554	3.26824719	0.00108216	0.01520216
COL12A1	3499.06866	1.223235272	0.38905381	3.14412874	0.00166582	0.02018377
COL13A1	29.6024059	-1.901205158	0.63631855	-2.98781981	0.00280975	0.02831477
COL17A1	41108.22	1.213216715	0.31282911	3.87820918	0.00010523	0.00323165
COL24A1	12.3430091	-1.644471591	0.55064167	-2.98646413	0.00282224	0.02838529
COL7A1	18618.6425	1.035350396	0.34583574	2.99376343	0.0027556	0.02787818
COLQ	14.3229278	-1.351019681	0.43383671	-3.11412025	0.00184494	0.02156233
CORO1A	300.414166	1.401572513	0.43562703	3.21736812	0.00129372	0.0170653
COTL1	4009.36804	1.159284779	0.34490988	3.36112374	0.00077626	0.01219759

CP	12271.1357	-1.986479497	0.71413625	-2.78165336	0.00540828	0.04361213
CPAMD8	186.613723	-2.28250425	0.27830326	-8.20150031	2.37E-16	1.84E-12
CPLX1	27.8488979	-1.338766429	0.48301501	-2.77168698	0.00557666	0.04451285
CREB3L1	888.082702	-1.986344378	0.46646781	-4.25826675	2.06E-05	0.00103402
CRISP3	135.024546	-5.104579959	1.54579849	-3.30222859	0.0009592	0.01403416
CRLF1	83.392321	-2.16956056	0.73655386	-2.94555587	0.00322375	0.03101559
CRTAC1	12.6091822	-2.131916002	0.73284986	-2.9090761	0.00362499	0.0335241
CRYM	138.04137	-2.646963903	0.89144533	-2.96929472	0.00298484	0.0295795
CSGALNACT1	259.572659	-2.466193609	0.56705182	-4.34915035	1.37E-05	0.00078808
CSPG4	1158.5661	1.971862842	0.5207749	3.78640146	0.00015284	0.0041057
CTGF	29082.1853	-1.603675553	0.39358333	-4.0745515	4.61E-05	0.00180559
CTNNAL1	1526.48479	1.290153542	0.32684224	3.9473281	7.90E-05	0.00264148
CXCL14	2452.83479	2.916194893	0.75567937	3.85903733	0.00011383	0.00340166
CXCL17	7434.62053	-1.509999076	0.47965705	-3.14808063	0.00164346	0.01999096
CXCR4	30.8181646	-2.695889944	0.88791935	-3.03618784	0.0023959	0.02573306
CYB5A	5036.98501	-1.301291487	0.43904756	-2.96389638	0.00303771	0.02982219
CYFIP2	1147.63017	-1.39631494	0.2728473	-5.11756918	3.09E-07	5.16E-05
CYP1A1	37.509759	-2.061531788	0.75640442	-2.72543594	0.00642166	0.04850257
CYP26B1	496.525518	2.061824786	0.5167184	3.99022911	6.60E-05	0.00233731
CYP2A6	250.042249	-4.697586383	0.95585978	-4.91451414	8.90E-07	0.00010784
CYP2B7P	2000.04852	-3.258092758	0.701111	-4.64704272	3.37E-06	0.00028188
CYP2F1	207.418035	-5.415785949	1.72599516	-3.13777586	0.00170235	0.02042163
CYP4B1	4589.28726	-2.674474433	0.92972036	-2.87664392	0.00401929	0.0355186
CYP4X1	2410.30813	-1.44221207	0.50110483	-2.87806457	0.00400123	0.03539938
CYP4Z1	29.7149681	-2.601382628	0.60710553	-4.28489367	1.83E-05	0.0009547
CYP7B1	76.793136	-1.914632821	0.4156671	-4.60616883	4.10E-06	0.00032239
CYS1	9.99817292	1.733104168	0.62860461	2.75706564	0.00583226	0.04568312
DBF4B	249.01585	1.129619714	0.33171152	3.40542802	0.0006606	0.01105212
DCDC2	54.0594957	-3.074843653	1.06247704	-2.89403304	0.00380328	0.03439566
DDIT4L	53.0960263	-1.740177196	0.48421137	-3.59383795	0.00032584	0.0068552
DEPDC7	130.970387	1.299921139	0.42304266	3.07278975	0.00212068	0.02367862
DFNA5	1921.12171	1.033361362	0.31583165	3.27187398	0.00106837	0.01507678
DFNB59	29.1796737	-1.221716838	0.32875687	-3.71617124	0.00020226	0.00489794
DMGDH	20.0519601	-3.789119845	0.96825574	-3.91334613	9.10E-05	0.00289287
DNAH1	103.782017	-1.071689204	0.32510011	-3.29648983	0.00097901	0.01421675
DNAH5	439.977388	-1.484339408	0.42407417	-3.50018818	0.00046493	0.00871898
DNAH7	11.7383082	-1.811348104	0.5661214	-3.1995754	0.0013763	0.01780239
DNM3	58.285858	1.375733716	0.44350892	3.10193021	0.00192263	0.02220262
DNMBP-AS1	14.6293979	-2.918045429	0.59307649	-4.92018392	8.65E-07	0.00010619
DOCK8	273.023944	-1.060941783	0.30780686	-3.44677757	0.00056732	0.00998694
DPF1	7.7207545	2.1478431	0.59856526	3.58831898	0.00033282	0.00693771
DSG3	10801.6252	1.57216014	0.44079364	3.56665796	0.00036156	0.00741191
DTL	412.989703	1.810086824	0.47399986	3.81874969	0.00013413	0.00378222
DUSP13	42.6466263	2.550500509	0.70436586	3.62098824	0.00029348	0.00641067
DUSP4	4541.80472	-1.409106209	0.3841112	-3.66848504	0.00024399	0.00560519
DUSP7	3817.50767	1.177359548	0.29763957	3.95565527	7.63E-05	0.00258456
DUSP8	209.182714	-2.03270175	0.52361576	-3.88204848	0.00010358	0.0032064
DYRK3	400.153591	1.172722265	0.26134534	4.48725143	7.21E-06	0.00049076
DZIP1	470.889176	1.648806449	0.55719052	2.95914303	0.00308496	0.03007274
E2F7	444.961133	1.814725826	0.31136063	5.82837279	5.60E-09	2.48E-06
EBF4	236.889023	-2.200038996	0.48526821	-4.53365576	5.80E-06	0.00040499
EDN1	3409.75111	-1.352311806	0.20150743	-6.71097725	1.93E-11	4.28E-08
EDN2	246.265329	-2.07781283	0.71851979	-2.89179626	0.00383046	0.03456292
EEPD1	158.469753	-1.064791658	0.32459434	-3.28037658	0.00103669	0.01476397
EFCAB4A	807.228314	-2.895953857	0.56919839	-5.08777593	3.62E-07	5.80E-05

EFCAB4B	99.5550322	-1.258477353	0.36678443	-3.43110899	0.00060112	0.01039326
EFHD1	670.371654	-4.615863147	0.65404559	-7.05740274	1.70E-12	6.49E-09
EGFL7	213.663955	1.068305548	0.32750681	3.2619338	0.00110655	0.01539146
EGR2	5674.83561	-1.701360466	0.61154892	-2.78205128	0.00540165	0.04358699
EGR4	124.70813	-1.996095968	0.66355919	-3.00816567	0.0026283	0.02708457
ELF5	168.621058	-1.988529239	0.60484168	-3.28768551	0.00101015	0.01451858
EMP3	281.770793	1.481141438	0.32574366	4.54695397	5.44E-06	0.00038721
ENPP3	71.5679111	-4.021915021	1.09075834	-3.68726499	0.00022668	0.0053029
ENPP4	379.195753	-1.728304791	0.47283283	-3.6552132	0.00025697	0.00578421
EPB41L2	1219.01374	1.231081107	0.40003817	3.07740911	0.00208809	0.02344454
EPHA1-AS1	6.85330409	2.166744114	0.63719084	3.40046338	0.00067272	0.01119439
EPHB6	534.291603	-1.42074936	0.47183628	-3.01110663	0.00260297	0.02693097
ERBB4	52.2019509	-2.142603308	0.50016662	-4.2837791	1.84E-05	0.00095628
ERN2	1497.24598	-2.024668775	0.61452831	-3.2946713	0.00098537	0.01429568
ERP27	37.4300297	-1.521795817	0.52069069	-2.92264844	0.00347068	0.0325631
ERRFI1	16604.7289	-1.206081505	0.29463218	-4.09351585	4.25E-05	0.00170271
ERV3-1	379.056992	-1.530533356	0.46540067	-3.28863592	0.00100674	0.01451074
ERVMER34-1	211.449475	1.135833958	0.31427826	3.61410288	0.00030139	0.00651009
ESRRG	63.0579484	-1.56837695	0.55576959	-2.82199132	0.00477265	0.03983799
ETNK2	1162.51363	1.089839519	0.23248026	4.68787987	2.76E-06	0.00024605
ETV4	230.701008	1.771917051	0.56939982	3.11190307	0.00185886	0.02164338
F8	153.892765	-1.079935794	0.3130008	-3.45026527	0.00056004	0.00990376
F8A1	272.931402	-1.190530451	0.22845735	-5.21117156	1.88E-07	3.54E-05
FABP5	859.70084	1.620589939	0.580825	2.79015183	0.00526833	0.04284561
FADS1	4942.42005	1.41550588	0.33449215	4.23180604	2.32E-05	0.00111656
FADS2	14472.362	1.262947972	0.41084219	3.07404641	0.00211177	0.0236472
FAM107B	3452.36569	-1.26041061	0.24687429	-5.10547534	3.30E-07	5.44E-05
FAM110B	64.0898103	-1.784867659	0.59261897	-3.01183009	0.00259678	0.02690233
FAM171B	453.938695	-1.090216868	0.32206953	-3.38503579	0.00071169	0.01161233
FAM174B	545.379515	-1.597111642	0.3618313	-4.41396766	1.01E-05	0.00062365
FAM189A2	302.049895	-1.128816914	0.41549271	-2.71681518	0.00659134	0.04933644
FAM213A	3512.82354	1.168692984	0.20633649	5.66401508	1.48E-08	4.94E-06
FAM221A	95.2196636	-1.579734774	0.40941158	-3.85854931	0.00011406	0.0034019
FAM222A	84.1998839	-1.566087553	0.53731951	-2.91462996	0.00356111	0.03315077
FAM46C	481.417544	-2.516091893	0.39273433	-6.40660039	1.49E-10	2.04E-07
FAM53A	23.665914	-1.487920851	0.41157472	-3.61519006	0.00030013	0.0065041
FAM83E	1636.10427	-1.416436112	0.49439762	-2.86497354	0.00417044	0.03628994
FANCB	50.965187	1.361852626	0.36013356	3.78152097	0.00015587	0.00414146
FANCD2	562.223348	1.125740158	0.38910862	2.89312573	0.00381429	0.03445297
FAT2	16127.4113	1.011676865	0.16954478	5.96701855	2.42E-09	1.29E-06
FBLN2	803.701882	-1.708482628	0.46270005	-3.6924194	0.00022213	0.00525156
FBLN5	219.463504	-2.191461077	0.61189649	-3.58142452	0.00034173	0.00710433
FBN2	1558.25466	2.031459268	0.69272213	2.93257453	0.00336164	0.03186527
FBXO17	374.290793	1.145273628	0.36570928	3.13165041	0.00173827	0.02064226
FBXO27	509.442502	1.48104182	0.34746257	4.26244998	2.02E-05	0.00102816
FCGBP	92.9458943	-2.387889748	0.58773594	-4.06286152	4.85E-05	0.0018842
FER1L6	853.123473	-2.251258832	0.70548701	-3.19107057	0.00141747	0.01808435
FGFBP1	22864.6218	1.779539625	0.43385484	4.10169359	4.10E-05	0.00166136
FGFBP3	24.1016449	-1.201545477	0.41947453	-2.86440627	0.00417792	0.03630027
FGFR2	3444.96714	-1.031251351	0.29635497	-3.47978423	0.00050182	0.00911322
FGFR4	79.8712389	1.498244321	0.43013057	3.48323142	0.0004954	0.00902839
FLJ22184	59.5973031	-1.888130516	0.5104579	-3.69889564	0.00021654	0.00516664
FMO5	123.017718	-2.682509514	0.49451116	-5.42456825	5.81E-08	1.43E-05
FMO6P	115.686188	-2.572902821	0.66262931	-3.88286902	0.00010323	0.00320202
FOLH1	44.5343485	-1.849848784	0.62144407	-2.9766939	0.00291375	0.02911682

FOLR1	1382.46613	-1.61353918	0.53282162	-3.02829147	0.00245941	0.02613107
FOXD2-AS1	120.173025	1.217887091	0.31499789	3.86633413	0.00011048	0.00334014
FOXE1	847.776037	1.937439701	0.45117154	4.29424179	1.75E-05	0.00093103
FOXI1	175.537737	-4.326497093	1.41229129	-3.06345945	0.00218794	0.02419066
FOXRED2	258.410942	-1.343952932	0.44283993	-3.03485036	0.00240655	0.0257935
FRMD4A	483.339509	1.396598097	0.45070729	3.09868094	0.00194384	0.02236088
FRY	303.845774	-2.122201504	0.61695853	-3.43977981	0.00058219	0.01016796
FSCN1	18487.7224	1.607966798	0.31213344	5.15153642	2.58E-07	4.61E-05
FSTL4	91.7976461	-1.752976409	0.53848465	-3.25538788	0.00113238	0.01562457
FUT2	2912.07685	-1.899793571	0.57908386	-3.28068817	0.00103554	0.01476123
FUT3	1195.63707	-1.380427162	0.4958702	-2.78384782	0.00537182	0.04343671
FXYD5	1355.61138	1.242971063	0.27514393	4.51753032	6.26E-06	0.00043318
FYB	2818.07122	1.522305987	0.46392773	3.28134294	0.00103314	0.01474055
FZD4	474.915849	-1.639225806	0.55174893	-2.97096329	0.00296867	0.02947576
FZD8	313.581334	-1.329558358	0.35542341	-3.74077318	0.00018346	0.00462635
GAL	93.0809379	2.565342006	0.79640584	3.22114917	0.00127678	0.01697118
GAL3ST4	163.542749	1.246299406	0.45263728	2.75341748	0.00589766	0.0459171
GATM	134.221079	-2.325009755	0.64655065	-3.59602107	0.00032312	0.00681748
GCNT3	2255.83291	-1.509708651	0.52823849	-2.85800577	0.00426313	0.03683389
GCSAM	52.1532155	1.321357698	0.34507022	3.82924289	0.00012854	0.00366401
GDF15	9406.14006	-2.078895471	0.5133565	-4.04961363	5.13E-05	0.00196942
GDPD1	152.767429	-1.426682021	0.23694186	-6.02123244	1.73E-09	1.12E-06
GGH	1585.47858	1.075178726	0.33550802	3.20462902	0.00135237	0.01764282
GINS2	244.498995	1.791952103	0.48223164	3.71595711	0.00020244	0.00489794
GINS4	359.988475	1.360068319	0.47111492	2.88691412	0.0038904	0.03484235
GJB2	20089.6091	1.585904062	0.52951644	2.99500437	0.00274441	0.02787818
GLIPR2	195.441455	-1.60706813	0.55249745	-2.90873401	0.00362895	0.0335408
GLRB	116.324442	-1.305601213	0.38688173	-3.3746779	0.00073902	0.01182815
GLTPD2	18.9439201	-1.661545166	0.42669665	-3.89397288	9.86E-05	0.00309601
GNA14	25.7863457	-3.028276152	0.84605808	-3.57927691	0.00034455	0.00713427
GOLM1	7100.46245	-1.277070978	0.2910716	-4.38748047	1.15E-05	0.00068401
GPLD1	57.4144335	-1.573016555	0.32787402	-4.79762482	1.61E-06	0.00016601
GPR116	907.793078	-2.42127493	0.81869624	-2.95747655	0.00310168	0.03018246
GPR143	51.5080631	-1.45753403	0.47043226	-3.09828674	0.00194643	0.02236088
GPR160	226.433086	-1.705523292	0.47218773	-3.6119602	0.00030389	0.00655501
GPR68	876.012077	1.461440268	0.33558544	4.35489773	1.33E-05	0.0007733
GPRIN2	106.79709	-1.764244569	0.6096184	-2.89401463	0.0038035	0.03439566
GPT	52.4463334	-3.406971006	0.78500689	-4.34005237	1.42E-05	0.00080924
GPX2	2057.1902	1.929226201	0.4323698	4.46198187	8.12E-06	0.00053113
GPX3	8849.55407	1.610921148	0.38499874	4.1842245	2.86E-05	0.00128631
GREM2	38.0473855	-3.446238996	1.00929843	-3.41448962	0.00063902	0.010843
GRID2IP	17.9921331	-1.502813716	0.40934583	-3.6712569	0.00024136	0.00556205
GRK5	256.136957	1.508926279	0.40865021	3.69246419	0.00022209	0.00525156
GSG2	160.051729	1.575710468	0.5263319	2.99375823	0.00275564	0.02787818
GSTA1	176.580647	-2.38550194	0.65895064	-3.6201527	0.00029443	0.00642237
GUCY1A3	457.226459	-2.395666979	0.65878255	-3.63650643	0.00027636	0.00608818
H2AFX	2733.25473	1.115178424	0.31149014	3.58014037	0.00034341	0.00712977
HAL	44.1257063	-1.797086946	0.6255647	-2.87274351	0.00406924	0.03571584
HCN2	37.8031758	-1.29731465	0.41532314	-3.12362721	0.00178637	0.02104298
HELLS	267.83553	1.339097576	0.41313225	3.24132911	0.00118974	0.01614691
HENMT1	117.767795	-1.586252766	0.54745781	-2.89748858	0.00376163	0.03427723
HEPACAM2	35.6423584	-6.646430748	1.50302056	-4.42204911	9.78E-06	0.00060664
HES6	219.335905	-2.142156577	0.4687091	-4.57033284	4.87E-06	0.00036308
HGD	51.9069262	-3.315292589	0.70206959	-4.72217093	2.33E-06	0.00021413
HID1	728.670725	-2.019204733	0.32282457	-6.2548049	3.98E-10	3.86E-07

HIF3A	35.584529	-2.37546158	0.85942726	-2.7640054	0.00570966	0.04516751
HIP1	1124.93425	1.009159121	0.28206877	3.57770593	0.00034662	0.00715816
HLA-DMA	375.273332	-1.087929548	0.3531402	-3.08072983	0.00206494	0.02327409
HLA-DRA	1179.59765	-2.126788935	0.76584778	-2.77703871	0.00548566	0.04401302
HMCN1	136.755306	1.815515425	0.32066073	5.66179541	1.50E-08	4.94E-06
HMGA1	16596.1628	1.397471462	0.28307353	4.93677899	7.94E-07	0.00010097
HMGA2	768.84201	2.201434594	0.44104887	4.99136201	6.00E-07	8.24E-05
HMGB2	2497.91751	1.437368968	0.45048362	3.19072417	0.00141917	0.01808534
HMGCS1	8285.73626	1.083844571	0.26931253	4.02448618	5.71E-05	0.00210347
HMOX1	529.22056	-1.726839479	0.44441323	-3.88566175	0.00010205	0.00317815
HMSD	31.1243861	1.550716572	0.51404344	3.01670336	0.0025554	0.02675354
HPX	20.4960564	-2.905710578	1.06810873	-2.72042583	0.00651979	0.0489663
HSD11B1	74.3849142	2.21816573	0.75082518	2.95430386	0.00313375	0.03039485
HSD11B2	1163.56675	-3.359092673	1.22362581	-2.74519599	0.00604748	0.0467549
HSD17B6	34.9589143	-1.951762471	0.68039138	-2.86858789	0.00412309	0.03604563
HSPA4L	2645.15107	1.029373692	0.2577471	3.99373525	6.50E-05	0.0023153
HUNK	57.6391031	-1.741713412	0.59045875	-2.94976307	0.00318018	0.03074898
ICA1	652.207024	-1.216297596	0.27605885	-4.40593588	1.05E-05	0.00063315
IDUA	535.366314	-1.013361298	0.30004941	-3.37731474	0.00073197	0.01175625
IGFBP5	4087.66802	-3.384718744	1.08272985	-3.12609718	0.00177143	0.02093987
IGFBP6	5718.01753	1.959782386	0.62487298	3.13628921	0.001711	0.02047528
IGSF3	6795.88055	1.087420774	0.2598697	4.1844846	2.86E-05	0.00128631
IKZF1	20.1583773	-1.873896028	0.61483333	-3.04781142	0.00230515	0.02503788
IL1R1	4371.75157	-1.533924587	0.40485608	-3.78881446	0.00015137	0.00408272
IL22RA1	111.176941	1.89877971	0.28277888	6.71471533	1.88E-11	4.28E-08
IL31RA	44.5494088	2.671170942	0.62432959	4.27846285	1.88E-05	0.00096993
IL34	31.5473881	-2.594697386	0.81595505	-3.17995139	0.001473	0.01858805
IL7R	30.8091372	1.800676054	0.5741941	3.13600582	0.00171266	0.02047927
IMPDH1	2944.3092	1.071510739	0.24297446	4.40997274	1.03E-05	0.00062844
INHBB	359.273963	-3.892847415	0.87196473	-4.46445514	8.03E-06	0.00052977
INO80C	1144.76178	1.346984141	0.26997594	4.98927467	6.06E-07	8.24E-05
INPP5J	194.953225	-2.055574458	0.4726425	-4.34911046	1.37E-05	0.00078808
IPCEF1	107.144584	-1.673686795	0.60580205	-2.76276187	0.00573146	0.04525855
IQCD	63.5576859	-1.520176566	0.55596267	-2.73431411	0.00625104	0.0476634
IQUB	8.7330697	-1.845963587	0.63446151	-2.90949658	0.00362011	0.03349901
IRX4	85.1332667	3.553748614	1.1974738	2.96770469	0.00300032	0.02967604
IRX6	13.3515933	-1.5504217	0.53703189	-2.8870198	0.0038891	0.03484235
ITGA6	19042.9995	1.400882095	0.24555419	5.70498146	1.16E-08	4.40E-06
ITGB5	3682.61424	1.004606405	0.27554275	3.64591852	0.00026644	0.0059456
ITIH4	27.1210298	-1.120993616	0.35175014	-3.18690312	0.00143805	0.01823607
IYD	82.9214095	-2.926560195	0.69862888	-4.18900545	2.80E-05	0.00127428
KAL1	1495.40802	-1.436912682	0.42909987	-3.34866727	0.00081201	0.01256836
KANSL1-AS1	21.8166504	-1.690434468	0.55751083	-3.03211053	0.0024285	0.02591915
KATNAL2	65.1124512	-1.537880939	0.36645347	-4.19666086	2.71E-05	0.00124661
KCNE3	75.8326775	-2.373773164	0.62794241	-3.78024026	0.00015668	0.00415318
KCNJ12	35.6897479	-2.311374808	0.79498398	-2.90744829	0.00364391	0.03365892
KCNK15	137.087892	-4.821273186	0.86463444	-5.57608277	2.46E-08	7.07E-06
KCNQ5	128.172328	2.181289863	0.54834025	3.97798603	6.95E-05	0.00241583
KDR	985.582755	-3.958640642	0.96164757	-4.11651916	3.85E-05	0.00159469
KIAA1199	1094.5689	1.527575675	0.51854207	2.94590501	0.00322011	0.03101559
KIAA1211L	436.697441	-1.403919426	0.39830755	-3.52471202	0.00042394	0.00821867
KIAA1524	534.167343	1.538849302	0.46975607	3.27584761	0.00105345	0.0149342
KIF11	1937.957	1.496550946	0.53711968	2.7862523	0.00533214	0.04313828
KIF13B	3211.35371	-1.081611076	0.29920237	-3.6149816	0.00030037	0.0065041
KIF20B	1598.32227	1.566655113	0.48567236	3.22574486	0.00125645	0.01681721

KIF22	1495.76149	1.028806633	0.33378771	3.08221845	0.00205464	0.02322552
KIT	301.726178	-3.911180149	1.2861096	-3.04109397	0.0023572	0.02542271
KLHDC7A	156.069527	-4.024598974	0.86486044	-4.65346635	3.26E-06	0.00027799
KLHDC7B	448.77433	-3.206213982	0.85318942	-3.75791578	0.00017133	0.00445097
KLHL24	1730.19057	-1.010158632	0.23551361	-4.28917301	1.79E-05	0.00094808
KLHL35	35.9044503	-2.238187775	0.64081646	-3.4927127	0.00047814	0.00884279
KLK13	1382.62578	-2.296593192	0.6727112	-3.41393629	0.00064032	0.01085317
KLK14	84.1403199	-3.601881378	0.64230906	-5.60770759	2.05E-08	6.23E-06
KLK5	246.2349	2.763558644	0.96885026	2.85241048	0.0043389	0.03726027
KLK9	140.501513	2.673003451	0.84364516	3.16839779	0.00153282	0.01917133
KLRAP1	18.5141351	-1.176853205	0.36084763	-3.26135768	0.0011088	0.01540895
KMO	45.4430126	-2.416318876	0.8395529	-2.87810198	0.00400076	0.03539938
KNTC1	711.842891	1.02181299	0.37186511	2.74780552	0.00599956	0.0464539
KREMEN1	4724.00181	1.049651239	0.18112745	5.79509764	6.83E-09	2.79E-06
KRT14	10656.7066	3.100378826	0.64292341	4.82231442	1.42E-06	0.0001539
KRT24	40.4415562	4.851749911	1.19055086	4.07521432	4.60E-05	0.00180501
KRT42P	53.2446622	1.446614454	0.52457017	2.75771389	0.00582071	0.04565963
KRT5	174438.174	1.426178738	0.33553647	4.25044331	2.13E-05	0.00105042
KRT6A	45828.0038	1.177885365	0.31298159	3.76343341	0.0001676	0.00439063
KRT74	10.4066923	2.195141187	0.73736814	2.97699489	0.00291089	0.02910701
KRT75	77.564021	-3.479299733	1.08187496	-3.21599062	0.00129995	0.01711455
LAMA1	264.695891	3.335951076	0.51794034	6.44080178	1.19E-10	2.04E-07
LAMA3	30321.8443	1.39392411	0.3619678	3.85096162	0.00011765	0.00344936
LAMC2	55294.8442	1.536773091	0.50517465	3.04206297	0.00234963	0.0253763
LBH	2615.41377	-1.562686721	0.45305476	-3.44922258	0.0005622	0.00991946
LCN2	6494.3057	-1.25427066	0.31580468	-3.97166583	7.14E-05	0.00244891
LDLRAD1	181.132296	-2.551824535	0.63046152	-4.04754999	5.18E-05	0.00197707
LDLRAD4	17.5803862	-1.778389225	0.64964933	-2.73746026	0.00619156	0.04734956
LEPREL1	818.403453	1.969010877	0.52849981	3.72566049	0.0001948	0.00481086
LGALS1	9449.33463	1.919081263	0.38017772	5.04785302	4.47E-07	6.48E-05
LGALS9B	39.297921	1.06144508	0.38208737	2.77801666	0.00546918	0.04394898
LIG1	870.154352	1.091115272	0.3038122	3.59141361	0.00032889	0.00690222
LINC00342	66.9048205	-1.873582752	0.38721826	-4.83857027	1.31E-06	0.00014384
LINC00346	31.8532167	-2.948308007	0.96862652	-3.04380268	0.00233608	0.02528284
LINC00669	39.8066463	1.930544916	0.4628154	4.1713066	3.03E-05	0.00134585
LINC00704	59.7642425	2.388093103	0.82597289	2.89124878	0.00383714	0.03457887
LINC00707	165.039943	1.635476679	0.55268724	2.95913593	0.00308503	0.03007274
LINC00887	16.0224724	3.267739893	0.88396875	3.69666903	0.00021845	0.00520413
LIPH	621.999663	-2.77896174	0.67288251	-4.12993604	3.63E-05	0.00152926
LIX1L	626.685175	1.038978026	0.29512976	3.52041096	0.00043088	0.00830872
LMNB2	6977.14818	1.046096851	0.25087696	4.16976052	3.05E-05	0.00135114
LMOD1	45.5060055	-3.018065707	0.95279146	-3.16760367	0.00153701	0.01918939
LOC100128653	25.451349	-1.855893748	0.64413746	-2.88120762	0.00396155	0.03520895
LOC100130938	17.7657223	3.049217439	0.75448235	4.04146953	5.31E-05	0.00200437
LOC100133445	24.1477681	-1.609362243	0.50488623	-3.18757404	0.00143472	0.0182087
LOC100288637	305.253676	1.510511142	0.54782034	2.75731117	0.00582789	0.0456719
LOC100505938	16.099517	-1.924439782	0.58196157	-3.30681591	0.00094363	0.01390121
LOC100506022	9.26565234	-1.479153631	0.48772316	-3.032773	0.00242318	0.0259047
LOC100506098	60.6072201	-2.221715383	0.48603787	-4.57107464	4.85E-06	0.00036308
LOC100506161	18.9767845	-1.175640548	0.36927297	-3.18366262	0.00145424	0.01838431
LOC100506411	329.909224	1.02689489	0.29998878	3.42311104	0.00061909	0.01059761
LOC100506731	38.5554488	1.580746641	0.548919	2.87974483	0.00397997	0.03534821
LOC100507336	72.1120855	1.383043156	0.46328876	2.9852724	0.00283326	0.02844079
LOC100507420	52.5594312	2.744028501	0.68052974	4.03219484	5.53E-05	0.00206837
LOC100507642	230.174833	-1.080340584	0.30958583	-3.48963194	0.00048369	0.00889856

LOC100507747	43.7539265	-4.433236702	0.69295778	-6.3975567	1.58E-10	2.04E-07
LOC100652768	33.4083036	-1.498256319	0.4426303	-3.38489328	0.00071206	0.01161233
LOC100996497	60.1600011	-1.198960038	0.41911548	-2.86069138	0.00422718	0.0366628
LOC101060376	39.715013	-4.52265091	0.87097103	-5.19265367	2.07E-07	3.78E-05
LOC101927181	71.0964428	-1.1952091	0.42066536	-2.84123491	0.00449392	0.0382316
LOC101927245	33.5522551	1.416439724	0.42680419	3.31871095	0.00090434	0.01361258
LOC101927377	15.8297739	1.792418699	0.608722	2.94456042	0.00323414	0.03109625
LOC101927841	105.968439	1.466430293	0.38760946	3.78326752	0.00015478	0.00413425
LOC101927910	17.5746453	-1.323064153	0.40560581	-3.26194575	0.0011065	0.01539146
LOC101928055	25.2468409	-1.243672998	0.45141837	-2.75503407	0.0058686	0.04585197
LOC101928123	75.8039877	1.204894916	0.38426939	3.13554746	0.00171534	0.0204955
LOC101928281	185.456753	-1.547876172	0.40915388	-3.78311497	0.00015488	0.00413425
LOC101928465	19.722573	-2.214854487	0.67115319	-3.30007295	0.0009666	0.0141025
LOC101928762	24.5111834	-1.240249845	0.32653205	-3.79824842	0.00014572	0.00397189
LOC101929479	50.2512525	-1.020827651	0.26653911	-3.82993569	0.00012818	0.00366401
LOC101930033	44.8939545	-1.637540546	0.40123362	-4.08126452	4.48E-05	0.00177665
LOC101930275	47.5226001	-1.933299812	0.47328609	-4.08484392	4.41E-05	0.00175397
LOC102659288	45.575399	-2.048622318	0.6233651	-3.28639236	0.0010148	0.01453247
LOC102723545	8.99726684	-4.791342623	1.44053339	-3.32608925	0.00088074	0.01337422
LOC102723602	155.310755	1.332038466	0.43324826	3.07453852	0.00210828	0.02362528
LOC102723629	37.0382434	3.34000434	0.74868051	4.46118779	8.15E-06	0.00053113
LOC102723721	80.5279761	1.612513813	0.45505714	3.54354142	0.00039479	0.00781969
LOC102724687	6.44152281	-1.707189134	0.59426486	-2.87277481	0.00406884	0.03571584
LOC158960	47.229079	-1.842225043	0.38596381	-4.77305129	1.81E-06	0.00018393
LOC283922	42.6529144	-1.06996862	0.3513841	-3.04501152	0.00232671	0.02521664
LOC613038	15.2232045	-1.755154554	0.52661936	-3.33287133	0.00085955	0.013095
LOC731656	20.6675582	-2.2720497	0.55461188	-4.0966481	4.19E-05	0.00168746
LPAR5	373.229228	1.463150875	0.44443159	3.29218469	0.00099412	0.0143745
LPHN1	1638.06221	-1.244249858	0.33291769	-3.73740989	0.00018593	0.00465334
LPPR3	23.94868	-3.458339597	1.06607131	-3.24400401	0.00117862	0.01607672
LRP8	577.663709	1.843166448	0.34587469	5.32900061	9.88E-08	2.25E-05
LRRC10B	16.3660494	-2.897414144	1.02994279	-2.81317969	0.00490542	0.04055341
LRRC16B	9.41413432	-1.881640307	0.66057505	-2.8484883	0.00439275	0.03755629
LRRC24	29.4605847	-1.068688214	0.34578542	-3.09061098	0.00199745	0.02281184
LRRC26	411.332321	-4.692049526	1.39613311	-3.36074654	0.00077732	0.0122019
LRRC46	12.6177577	-2.04963028	0.62354021	-3.287086	0.0010123	0.01452336
LRRC6	54.3375515	-1.024687151	0.34926419	-2.93384546	0.00334791	0.03175701
LRRC8C	1532.71034	1.194147295	0.33309602	3.58499416	0.00033709	0.00701727
LTB4R	1516.04007	1.385145702	0.31774966	4.3592358	1.31E-05	0.00076385
LTB4R2	473.074013	1.428513105	0.36390875	3.92547065	8.66E-05	0.00280155
LTF	763.354393	-4.870374504	1.02175075	-4.7666953	1.87E-06	0.00018583
LURAP1L	1690.70759	-1.080677593	0.31795996	-3.39878511	0.00067686	0.01123919
LY6D	2452.72688	2.456665738	0.69973131	3.5108701	0.00044664	0.00853076
LYPD1	151.098038	-2.502636979	0.6841823	-3.65785111	0.00025434	0.00574169
LYZ	18.7210795	-3.076602693	0.79942886	-3.84850089	0.00011884	0.00346454
LZTS3	1810.27524	-1.772437991	0.34870372	-5.08293394	3.72E-07	5.88E-05
MACROD2	337.948769	-3.307236345	0.74979778	-4.41083773	1.03E-05	0.00062844
MAD2L1	882.634377	1.5131882	0.50450472	2.99935388	0.00270553	0.02764166
MAFB	732.658353	-1.511530745	0.4728164	-3.19686614	0.00138929	0.01791069
MAFK	3281.79338	-1.006742419	0.34224725	-2.94156468	0.00326559	0.03129948
MAGI2-AS3	219.374793	1.418155567	0.46945838	3.02083342	0.0025208	0.02652313
MAMDC2	76.9976905	1.975881404	0.54937489	3.59659941	0.0003224	0.00681748
MAN1C1	936.40881	-1.396445566	0.43398262	-3.21774536	0.00129202	0.0170653
MANEAL	93.9319773	-2.26442992	0.49907967	-4.53721132	5.70E-06	0.00040003
MAP3K8	865.617835	-1.221059544	0.29115751	-4.19381086	2.74E-05	0.00125493

MAP6	25.9356868	-1.722508328	0.46083567	-3.73779301	0.00018564	0.00465334
MARVELD1	4701.1482	1.281850349	0.26514964	4.83444127	1.34E-06	0.00014583
MATN2	408.489913	1.645087098	0.46416073	3.54421863	0.00039378	0.00780961
MB	172.403465	-2.994737462	0.76080236	-3.93628835	8.28E-05	0.00271905
MBNL1-AS1	135.20864	-1.405682201	0.45985764	-3.05677688	0.00223731	0.02460879
MCAM	4027.14801	1.660985354	0.48515418	3.4236237	0.00061792	0.01059761
MCF2L	216.834753	-4.011028148	0.67484204	-5.9436548	2.79E-09	1.35E-06
MCIDAS	117.431719	-1.749983336	0.44362071	-3.94477375	7.99E-05	0.00264932
MCM2	1274.94932	1.285312382	0.32812329	3.91716285	8.96E-05	0.00287694
MCM5	1324.08101	1.323316713	0.39008186	3.39240775	0.00069281	0.01138224
MCM6	1205.95271	1.300007382	0.39213013	3.31524485	0.00091563	0.01372555
MDGA1	179.066459	2.181202851	0.79334196	2.74938547	0.00597071	0.04634624
MEIS3P1	305.316393	-1.329782761	0.27639753	-4.81112389	1.50E-06	0.00016053
MELK	955.758235	1.544800701	0.48296738	3.19856113	0.00138115	0.01782241
MESP1	102.446389	-3.499921649	0.7387865	-4.73739255	2.16E-06	0.00020309
MFAP3L	102.557595	-1.454365255	0.35063419	-4.14781363	3.36E-05	0.00145009
MFAP5	26.9267055	2.394652429	0.75906101	3.15475618	0.00160632	0.01970923
MFNG	22.291075	2.204261717	0.63664355	3.46231688	0.00053555	0.00959708
MFSD2A	1517.5039	1.093702711	0.37844259	2.89000959	0.0038523	0.03465507
MFSD4	279.312941	-2.897002608	0.63042356	-4.59532729	4.32E-06	0.00033673
MFSD6L	21.8573655	-3.540140854	0.96020468	-3.68686067	0.00022704	0.0053029
MFSD7	197.056831	-1.691578563	0.46208331	-3.66076532	0.00025146	0.00571938
MGAM	43.7665217	-2.80776703	0.53003288	-5.29734501	1.17E-07	2.60E-05
MGAT4A	492.810324	-1.586179698	0.5402195	-2.9361763	0.00332285	0.03172001
MGC32805	16.9675359	-2.656595538	0.82356247	-3.22573651	0.00125649	0.01681721
MIR21	35.1374695	-1.409391178	0.37843843	-3.72422848	0.00019591	0.00482239
MIR3189	11.2727485	-2.901173452	0.61424852	-4.72312647	2.32E-06	0.00021413
MIR503HG	19.5236495	-2.542579582	0.75554657	-3.36521888	0.00076483	0.01209148
MLPH	950.972268	-2.066933551	0.48524378	-4.25957761	2.05E-05	0.00103132
MMP15	2053.81973	-1.225650821	0.24988377	-4.90488371	9.35E-07	0.00011067
MORN3	9.13185167	-1.455722026	0.50424738	-2.88692036	0.00389033	0.03484235
MPZ	18.2555467	2.379445906	0.53209145	4.47187396	7.75E-06	0.0005161
MRAS	466.882762	-1.366443138	0.39327932	-3.47448507	0.00051183	0.00923029
MSLN	4566.029	-2.488361331	0.64322495	-3.8685709	0.00010948	0.00332912
MSN	15638.4729	1.493218521	0.28047785	5.32383753	1.02E-07	2.28E-05
MUC16	2145.75287	-4.825747363	0.94051711	-5.13095116	2.88E-07	4.97E-05
MUC20	3038.69961	-1.775000618	0.56286912	-3.15348728	0.00161332	0.01974087
MUC5AC	229.406813	-3.229412148	1.00072261	-3.22708024	0.0012506	0.01681721
MUC5B	2884.01172	-3.9377355	1.12578731	-3.4977615	0.00046918	0.00878195
MYH16	26.4733987	2.330694677	0.82099526	2.83886496	0.00452743	0.03839934
MYLIP	482.032865	-1.0599623	0.28270484	-3.74936029	0.00017729	0.00453718
MYLPF	7.89547062	-3.269650974	0.93655197	-3.49115806	0.00048093	0.0088584
NAGS	211.854367	1.236392749	0.32055614	3.85702401	0.00011478	0.00341073
NAV3	45.7827577	1.923722078	0.69233171	2.77861326	0.00545915	0.0438911
NBEA	355.149154	-1.804834969	0.4639335	-3.89028805	0.00010013	0.00313706
NCAPD2	4972.41642	1.260898874	0.37016338	3.4063307	0.00065842	0.01104751
NCAPG2	1303.4039	1.312545624	0.40791907	3.2176618	0.0012924	0.0170653
NCF1	43.9497443	2.23474216	0.53086177	4.20964982	2.56E-05	0.00119839
NCF1B	14.5331087	1.601164002	0.45643029	3.50801437	0.00045146	0.00857838
NCF1C	23.8511669	2.133218886	0.53289828	4.00305077	6.25E-05	0.0022397
NCMAP	122.425649	-2.502997897	0.5759935	-4.34553151	1.39E-05	0.00079807
NCOA7	26553.9203	-1.095612904	0.26527293	-4.13013452	3.63E-05	0.00152926
NDRG2	2086.15424	-1.669253289	0.53069113	-3.14543278	0.00165841	0.02010972
NEB	13.0266327	-1.956355026	0.70559967	-2.7726133	0.00556082	0.04443209
NEBL	2652.81917	-1.479298932	0.411403	-3.59574175	0.00032347	0.00681748

NECAB3	703.744314	-1.26671314	0.36361627	-3.48365366	0.00049462	0.00902839
NEFM	9.23735874	1.955056722	0.63206128	3.09314426	0.00198048	0.02265754
NEK5	8.20634763	-2.317958117	0.60104854	-3.85652401	0.00011501	0.00341073
NIPAL4	611.211676	1.92309563	0.49931041	3.85150318	0.0001174	0.00344936
NKD1	152.675981	-2.024695385	0.71463068	-2.83320525	0.00460838	0.03884312
NOS1	46.0186697	4.133239597	1.14773922	3.60120099	0.00031675	0.00674792
NPR2	177.775904	1.009510684	0.34588093	2.91866534	0.00351533	0.03284493
NPTXR	216.484078	-1.866318577	0.68814663	-2.71209434	0.00668596	0.04978036
NR3C2	858.281857	-2.531868767	0.68342888	-3.70465578	0.00021168	0.00508979
NR4A2	2088.91006	-1.264658253	0.40201664	-3.14578581	0.00165641	0.02010118
NRCAM	231.79968	3.02200729	0.74409784	4.06130369	4.88E-05	0.00189208
NRG1	1039.71531	1.525722891	0.53235085	2.86601003	0.00415681	0.03621796
NRG2	61.5649893	-2.182472449	0.63038147	-3.46214562	0.00053589	0.00959708
NRP2	2719.65017	1.012493852	0.3373138	3.00163781	0.00268531	0.02750763
NRXN3	277.262396	-1.872873748	0.55430324	-3.3787891	0.00072806	0.01172529
NT5E	1826.54485	2.683988291	0.66948941	4.00900784	6.10E-05	0.00220432
NUP62CL	74.2859983	1.267372955	0.23968338	5.2876965	1.24E-07	2.69E-05
OAS3	3657.17743	1.506073265	0.53936166	2.79232539	0.00523307	0.04262589
OLFM4	178.662109	-6.338183385	1.41527437	-4.47841319	7.52E-06	0.00050708
OPLAH	1084.63202	-1.317672316	0.39781539	-3.31227085	0.00092542	0.01381359
ORC1	267.955296	2.066837102	0.61260786	3.37383379	0.00074129	0.01184023
ORC6	282.63123	1.096874547	0.36741706	2.9853664	0.00283239	0.02844079
OSR1	174.967592	-1.728933786	0.53502593	-3.23149531	0.00123144	0.01662181
P2RY1	577.011368	1.043121823	0.32974165	3.1634518	0.0015591	0.01942177
PADI2	918.845197	-2.933159975	0.79606906	-3.68455466	0.0002291	0.00534309
PAK3	30.358164	2.206371085	0.74447659	2.96365407	0.0030401	0.02982219
PANX2	192.181337	2.188672904	0.5154305	4.24630071	2.17E-05	0.00106327
PARD3B	277.489845	-1.313677684	0.34471633	-3.8108948	0.00013846	0.00384431
PARD6A	65.6563455	-1.245535026	0.42857281	-2.90623905	0.00365802	0.03370898
PARM1	514.604682	-1.940678638	0.61461877	-3.15753232	0.00159111	0.01966024
PC	3762.62968	1.259606025	0.3493685	3.60537954	0.0003117	0.00666774
PCDHA10	8.07240035	-2.253421472	0.77971001	-2.89007637	0.00385148	0.03465507
PCDHA11	16.2359254	-2.258716402	0.75918222	-2.97519667	0.00292801	0.02920287
PCDHA12	55.0510626	-3.014684204	0.70891803	-4.25251452	2.11E-05	0.00104406
PCDHA4	38.9528361	-2.431334157	0.48061475	-5.05880049	4.22E-07	6.29E-05
PCDHA6	15.0308242	-2.96811578	1.07848141	-2.75212513	0.00592099	0.04602938
PCDHAC1	72.7737942	-3.749714131	0.65843099	-5.69492354	1.23E-08	4.45E-06
PCDHB10	45.4836062	-1.984885508	0.47140651	-4.21056022	2.55E-05	0.00119719
PCDHB13	51.9237203	-2.539903975	0.66624082	-3.81229116	0.00013768	0.00384056
PCDHB14	104.397339	-1.99356399	0.5265445	-3.78612632	0.00015301	0.0041057
PCDHB15	30.5097771	-1.377173358	0.48358099	-2.84786499	0.00440136	0.0375787
PCDHB16	31.2877676	-2.088644453	0.49910935	-4.18474317	2.85E-05	0.00128631
PCDHB2	94.5470201	-2.264818926	0.49010797	-4.62106124	3.82E-06	0.00030839
PCDHB4	57.5290595	-2.195364284	0.41115158	-5.33954972	9.32E-08	2.16E-05
PCDHB5	145.792745	-3.078478101	0.58391874	-5.27210023	1.35E-07	2.83E-05
PCDHB6	36.6790257	-3.259309408	0.8598149	-3.79071054	0.00015022	0.00408006
PCDHB7	55.2801216	-2.477733455	0.6402095	-3.87019166	0.00010875	0.00331356
PCDHB8	60.012005	-3.398181982	1.05945796	-3.20747223	0.00133907	0.01751065
PCDHGA11	93.5464425	1.074489111	0.2421725	4.43687496	9.13E-06	0.00057778
PCDHGA2	62.7210853	-2.434002847	0.69202083	-3.51723928	0.00043606	0.00836988
PCDHGA3	14.2077965	-2.774187227	0.73008129	-3.79983333	0.00014479	0.00396749
PCDHGA6	31.7883542	-1.673085537	0.4939862	-3.38690741	0.00070685	0.01155171
PCDHGA7	28.5766505	-1.913311857	0.55874841	-3.4242815	0.00061643	0.01058712
PCDHGB1	22.6949562	-2.043338187	0.71091602	-2.87423288	0.0040501	0.03568922
PCDHGB2	40.9622762	-1.403015032	0.48981061	-2.86440308	0.00417796	0.03630027

PCDHGB3	21.0690155	-1.652372498	0.45669965	-3.61807265	0.00029681	0.00645603
PCP2	15.4152335	-2.848904372	0.66247942	-4.30036663	1.71E-05	0.00092487
PCSK6	413.306578	-2.081517448	0.50571985	-4.11594969	3.86E-05	0.00159469
PCSK9	1878.39154	1.926613422	0.5608636	3.43508372	0.00059237	0.01029942
PCYT1B	49.1969509	2.987555574	0.46985579	6.35845213	2.04E-10	2.26E-07
PDCL3P4	22.5635421	-1.448567822	0.42463616	-3.41131528	0.0006465	0.01091035
PDE11A	21.161218	-2.392864784	0.74181976	-3.2256687	0.00125679	0.01681721
PDE4C	38.5107247	-2.625541223	0.82592675	-3.17890326	0.00147833	0.01863621
PDE7B	45.4032609	-1.941875239	0.6690236	-2.90255119	0.00370137	0.03394707
PDE8B	125.139166	-5.770561197	0.82106406	-7.02814982	2.09E-12	6.49E-09
PDGFD	109.227181	-1.609017145	0.44339437	-3.62886234	0.00028467	0.00623587
PDK2	562.916984	-1.243627971	0.33810596	-3.67821964	0.00023487	0.00545294
PDK4	718.649121	-3.171225469	0.57552562	-5.51013775	3.59E-08	9.76E-06
PDLIM4	1560.74683	1.117015138	0.31654607	3.52876014	0.00041751	0.00814298
PEAR1	541.729049	2.516330415	0.56806246	4.42967206	9.44E-06	0.00059499
PGBD5	133.80758	-2.365559553	0.78418782	-3.01657268	0.0025565	0.02675354
PGPEP1	674.341793	-1.060352657	0.21301722	-4.97777895	6.43E-07	8.45E-05
PHACTR3	318.23669	-1.687631978	0.55509086	-3.04028061	0.00236358	0.02547376
PHF19	818.461352	1.51390633	0.29322164	5.16301018	2.43E-07	4.38E-05
PIGR	8364.74275	-3.232721524	0.56842829	-5.68712288	1.29E-08	4.45E-06
PIK3R3	1716.14698	-1.192541789	0.25981711	-4.58992784	4.43E-06	0.00034079
PIP	129.496525	-3.832239735	0.82500332	-4.6451204	3.40E-06	0.00028188
PIP5K1B	131.187026	-2.396257519	0.72890629	-3.28746993	0.00101092	0.01451858
PITX1	5516.04522	1.286945023	0.38320782	3.35834748	0.0007841	0.01227104
PKMYT1	693.333468	1.793037087	0.6066231	2.9557679	0.00311892	0.03028884
PLCD3	5233.25489	1.090991386	0.21901179	4.98142754	6.31E-07	8.44E-05
PLEK2	1229.96564	1.471652948	0.3586468	4.10334886	4.07E-05	0.0016576
PLEKHB1	96.5709972	-2.141015588	0.65396883	-3.27388018	0.00106082	0.01500394
PLEKHG7	120.360382	-2.198067023	0.55326222	-3.97292089	7.10E-05	0.00244143
PLEKHS1	3781.43074	-2.350223631	0.66612894	-3.52818123	0.00041843	0.00814298
PLK4	524.302487	1.519452334	0.50034254	3.03682417	0.00239085	0.02571406
PNPLA7	41.2731222	-2.135632991	0.44429266	-4.80681585	1.53E-06	0.00016234
POC1A	385.223979	1.115200683	0.40785272	2.73432203	0.00625089	0.0476634
POLN	16.2620938	-2.127342425	0.52872905	-4.02350209	5.73E-05	0.00210662
POLQ	313.18739	1.841983079	0.54613281	3.372775	0.00074415	0.01186123
PPARGC1A	1216.16754	-1.164813757	0.38148874	-3.05333721	0.00226311	0.02468259
PPM1H	732.279335	-2.153727177	0.61951073	-3.47649695	0.00050801	0.00917198
PPP1R16B	599.607082	-3.252432369	0.71328512	-4.55979282	5.12E-06	0.00037202
PPP1R9A	248.306917	-2.965917016	0.5841878	-5.0769924	3.83E-07	6.01E-05
PPP4R4	598.545955	1.017288437	0.28979653	3.51035405	0.00044751	0.00853146
PRKAR2B	161.240217	-2.377124183	0.69234185	-3.43345444	0.00059594	0.01032678
PRKCDBP	1519.61475	1.233545431	0.27976378	4.40923917	1.04E-05	0.00062844
PROB1	125.352275	1.264217172	0.30103206	4.19960976	2.67E-05	0.00123858
PROCR	507.156848	1.942167862	0.35779554	5.42815005	5.69E-08	1.42E-05
PROM1	273.185159	-4.045081858	0.75721159	-5.342076	9.19E-08	2.16E-05
PRR11	2426.85352	1.94549593	0.62155952	3.13002356	0.00174792	0.02072518
PRR15L	545.923137	-2.111947327	0.60549324	-3.48797839	0.00048669	0.00894317
PRR22	39.5273176	-1.058167694	0.36532844	-2.89648318	0.00377371	0.03432636
PRSS2	145.66986	-3.672841977	1.06805347	-3.43881845	0.00058426	0.01019266
PRSS3	188.168335	2.697675008	0.68859933	3.91762653	8.94E-05	0.00287694
PSMC3IP	138.262634	1.024972785	0.27555622	3.71965036	0.0001995	0.00488017
PSMG3-AS1	230.784006	-1.122587767	0.29372914	-3.82184675	0.00013246	0.00374182
PSRC1	469.221908	1.621825975	0.55443898	2.92516585	0.00344273	0.03239882
PTPLAD2	689.156741	1.018171804	0.29770366	3.42008499	0.00062602	0.0106926
PTPN22	11.4072219	-2.157807844	0.75707677	-2.85018366	0.0043694	0.03745993

PTTG1	2026.10156	1.60213843	0.52022339	3.07971239	0.00207201	0.02330293
PXMP4	1320.17288	-1.842283989	0.48747493	-3.77923845	0.00015731	0.00415622
PYCARD	1485.05581	1.044112301	0.25170734	4.14812017	3.35E-05	0.00145009
RAB11FIP1	7306.65765	-1.495285601	0.51113567	-2.92541822	0.00343994	0.03239882
RAB17	168.924198	-2.712797704	0.48337787	-5.61216777	2.00E-08	6.20E-06
RAB19	32.5528361	-1.954255273	0.49118921	-3.97862013	6.93E-05	0.00241583
RAB37	173.006571	-2.517802129	0.70692888	-3.56160599	0.00036859	0.00751803
RAC2	2736.89695	1.746626309	0.35864318	4.87009491	1.12E-06	0.00012536
RAD51	264.675967	1.787404567	0.47267902	3.78143411	0.00015593	0.00414146
RALGPS1	256.58589	-1.519631855	0.36297059	-4.18665282	2.83E-05	0.00128379
RARRES1	4372.84	-3.286395257	0.95644745	-3.43604372	0.00059028	0.01027451
RASD1	1441.09727	-2.105688311	0.4565086	-4.61259288	3.98E-06	0.00031792
RASD2	66.1379083	-2.075555844	0.67887218	-3.05735881	0.00223297	0.02460764
RASGEF1B	291.039603	-1.365447955	0.43040477	-3.17247404	0.00151146	0.01896541
RBP1	1049.23734	1.221913949	0.44850546	2.72441264	0.0064416	0.04853945
RCAN2	37.3609465	-3.912848905	0.90605572	-4.31855216	1.57E-05	0.00086375
RDH10	6654.65305	-1.707255178	0.46295399	-3.68774263	0.00022625	0.0053029
RECQL4	539.33992	1.245363238	0.38662745	3.2210937	0.00127702	0.01697118
REEP2	197.374284	1.729961341	0.61581635	2.80921634	0.00496623	0.0410342
REEP6	410.390152	-1.024759309	0.35903794	-2.85418113	0.00431479	0.03711487
REPS2	618.982581	-1.014553096	0.34709079	-2.92301931	0.00346655	0.03254458
RERG	71.7736431	-3.109215894	0.78894995	-3.94095453	8.12E-05	0.00267804
RGL3	35.7042713	-2.731288444	0.96769129	-2.8224791	0.00476539	0.0398203
RGS10	634.295638	1.007249858	0.29146558	3.45581067	0.00054864	0.00975787
RGS20	423.832798	1.069418036	0.27830474	3.84261531	0.00012173	0.00352457
RHCG	1784.44045	2.402825028	0.60914877	3.94456187	7.99E-05	0.00264932
RHOU	242.934767	-1.977032655	0.68563019	-2.88352626	0.0039325	0.03503109
RHOV	6383.72366	-1.388674601	0.34523372	-4.02241879	5.76E-05	0.00210701
RIC3	78.2892285	-1.603017224	0.48267862	-3.32108607	0.00089668	0.01352781
RIMS1	40.6587061	-2.826770877	0.84958907	-3.32722133	0.00087717	0.01333723
RIMS2	107.684068	2.058542089	0.43187476	4.76652556	1.87E-06	0.00018583
RIPPLY3	24.9434104	1.52612325	0.52261454	2.92016993	0.00349841	0.03276375
RMI2	349.36794	1.667868447	0.35341646	4.71927208	2.37E-06	0.00021593
RNASE6	7.24191087	-2.967503669	1.0082034	-2.94335811	0.00324673	0.03117862
RNF208	171.834164	-1.115971317	0.28752383	-3.88131771	0.00010389	0.0032064
RNF223	113.335359	-2.201429838	0.59928115	-3.67345085	0.0002393	0.0055227
RNR1	40300.4859	-1.466942435	0.28968985	-5.0638379	4.11E-07	6.29E-05
RNR2	360691.385	-1.079708399	0.29441394	-3.66731411	0.00024511	0.00560519
RORC	270.860506	-1.986938413	0.45769988	-4.34113819	1.42E-05	0.00080924
RP1-27K12.2	21.4424985	1.558775216	0.5494832	2.83680233	0.00455678	0.0385549
RP11-132A1.4	49.0627622	2.232256594	0.35050338	6.36871629	1.91E-10	2.26E-07
RP11-21L23.3	8.5635534	1.474146476	0.53503865	2.75521493	0.00586536	0.04585197
RP11-294O2.2	123.063491	-3.702402326	0.66363712	-5.57895603	2.42E-08	7.07E-06
RP11-37B2.1	10.4802567	-2.401951882	0.54882566	-4.37652983	1.21E-05	0.00071108
RP11-395P17.3	85.6385542	-1.135470347	0.27795855	-4.08503483	4.41E-05	0.00175397
RP11-75C9.1	13.489724	1.973314977	0.67249273	2.9343291	0.0033427	0.03175701
RP11-92G12.3	8.50093381	2.239785146	0.58182509	3.84958502	0.00011832	0.00345574
RP3-395M20.9	12.1401082	-2.628491753	0.92624665	-2.83778813	0.00454273	0.03847801
RP5-1159O4.2	11.2447685	-2.722525534	0.86228623	-3.15733388	0.00159219	0.01966024
RPP25	522.904518	-1.069178491	0.35654444	-2.99872438	0.00271112	0.02766239
RPS6KA2	2369.53564	-1.914912526	0.39299151	-4.87265622	1.10E-06	0.00012465
RPSAP52	55.2688399	3.381857672	0.90480578	3.73766145	0.00018574	0.00465334
RRAD	295.806394	1.889350675	0.66097108	2.85844683	0.0042572	0.03680322
RSPH4A	13.4572537	-2.471007247	0.50652459	-4.87835594	1.07E-06	0.00012264
RTN4R	318.080627	1.128113203	0.33152009	3.40285016	0.00066687	0.01112093

RUNX3	272.740506	1.676117931	0.5487286	3.05454815	0.002254	0.02468259
S100A14	22593.3025	1.117413198	0.30282068	3.69001611	0.00022424	0.00528531
S100A2	73145.5875	1.184593221	0.35191068	3.36617581	0.00076218	0.01208704
SAMD10	242.408857	-1.049745425	0.31167985	-3.36802468	0.00075709	0.01203041
SAMD12	1542.09237	-1.009478061	0.32755769	-3.08183287	0.0020573	0.02323355
SAMD12-AS1	13.4549502	-2.024771539	0.65031775	-3.11351111	0.00184876	0.0215833
SARM1	152.945696	-2.034009908	0.49058967	-4.14605126	3.38E-05	0.0014532
SATB2	142.698528	-1.191881583	0.31326839	-3.80466597	0.000142	0.00391156
SBK1	164.625213	-2.167809102	0.44057669	-4.92038988	8.64E-07	0.00010619
SCARF1	41.0325101	-2.104184396	0.39199246	-5.36792062	7.96E-08	1.90E-05
SCD	59739.4807	1.52557081	0.36069602	4.22951941	2.34E-05	0.00112197
SCD5	2033.38551	-1.067998098	0.31730099	-3.3658833	0.00076299	0.01208704
SCGB1A1	11372.4435	-3.054703486	0.69645429	-4.38607896	1.15E-05	0.0006858
SCGB3A1	4858.44167	-4.859059668	0.75802163	-6.41018604	1.45E-10	2.04E-07
SCXB	81.1861241	-1.090485207	0.3534152	-3.08556396	0.00203167	0.02313443
SEMA3D	61.0468168	1.941858478	0.51959208	3.73727499	0.00018603	0.00465334
SEMA3E	73.7725541	-1.55253115	0.56059299	-2.76944448	0.0056152	0.04470539
SERHL2	139.102338	-3.001757868	0.92474248	-3.24604736	0.00117019	0.01601812
SERPINA3	42270.3798	-1.884568587	0.62522316	-3.01423349	0.00257629	0.02679795
SERPINB5	20309.336	1.010801371	0.34202253	2.95536485	0.00312299	0.03030947
SERPINE2	1227.59782	1.407191167	0.51291801	2.74350118	0.00607878	0.0468002
SFN	38473.0401	1.152933086	0.27011899	4.26824147	1.97E-05	0.00100844
SGK1	4275.05821	-1.026675846	0.30402958	-3.37689463	0.00073309	0.01175751
SGSM1	21.813341	-2.665857046	0.85123956	-3.13173537	0.00173776	0.02064226
SH3BGR	69.3432024	-1.067279976	0.3773333	-2.82848075	0.00467695	0.03931428
SH3BGRL2	1232.17564	-1.177264419	0.35573561	-3.30938027	0.00093503	0.01385693
SH3BP1	3915.93652	1.436089326	0.24108706	5.95672508	2.57E-09	1.33E-06
SH3PXD2A-AS1	36.3061738	1.877479505	0.62812291	2.98903204	0.00279863	0.02825776
SH3TC1	1708.94955	1.150811503	0.24572382	4.68335352	2.82E-06	0.00025011
SHC2	176.340851	-2.956796886	0.66341517	-4.45693289	8.31E-06	0.00053503
SKA1	309.580462	1.846457763	0.61220263	3.01608924	0.00256058	0.02676677
SKP2	876.663479	1.017234631	0.3128871	3.2511236	0.0011495	0.01577662
SLAIN1	113.15108	-2.449558569	0.76549042	-3.19998593	0.00137434	0.01780217
SLAMF9	18.0151367	2.606440288	0.89309157	2.91844687	0.0035178	0.03284493
SLC10A6	54.6428399	2.406202111	0.67544738	3.56238275	0.0003675	0.00750938
SLC16A1-AS1	30.1983408	1.43737971	0.52044206	2.76184385	0.0057476	0.04527191
SLC16A11	40.9291355	-1.717593169	0.54371544	-3.15899285	0.00158315	0.01962932
SLC16A6	24.8326264	-3.059552001	0.870273	-3.51562328	0.00043872	0.00841058
SLC1A1	191.367714	-1.821081673	0.47701805	-3.8176368	0.00013474	0.00379242
SLC23A3	10.1968242	-1.533063619	0.54401199	-2.81806957	0.00483133	0.04024122
SLC26A11	274.265438	-1.263900071	0.29541453	-4.27839512	1.88E-05	0.00096993
SLC27A2	214.462294	-1.661695186	0.52915465	-3.1402827	0.00168785	0.02033659
SLC2A11	148.857624	-1.388252482	0.28221823	-4.91907438	8.70E-07	0.00010619
SLC30A2	13.8559894	-2.754988865	0.81159398	-3.3945408	0.00068744	0.01134199
SLC34A2	17935.53	-3.088749024	0.65969232	-4.68210551	2.84E-06	0.00025021
SLC35E2	438.601546	-1.84951111	0.37152652	-4.97814018	6.42E-07	8.45E-05
SLC41A2	880.926377	-1.519895367	0.35350161	-4.29954297	1.71E-05	0.00092487
SLC43A2	3763.35381	-1.362572822	0.39633317	-3.43794802	0.00058614	0.01021399
SLC44A4	4115.45434	-1.57860587	0.4039072	-3.90833801	9.29E-05	0.00293544
SLC45A4	2026.22781	-1.104830932	0.37566034	-2.9410369	0.00327116	0.03131627
SLC46A3	968.08617	-2.214811178	0.36933003	-5.99683473	2.01E-09	1.15E-06
SLC5A5	2270.22658	-3.558386595	0.71336958	-4.98813899	6.10E-07	8.24E-05
SLC6A15	295.397557	2.216478033	0.46132677	4.80457278	1.55E-06	0.00016251
SLC9A2	83.4105655	-4.210006669	1.05074148	-4.00670072	6.16E-05	0.00221587
SLC9A7P1	17.8769211	-2.378657299	0.73740325	-3.22572121	0.00125656	0.01681721

SLFN11	498.259394	3.104230653	0.50961919	6.09127506	1.12E-09	7.90E-07
SLPI	35466.1802	-1.681008969	0.49494867	-3.39632993	0.00068296	0.01128896
SMIM2-AS1	20.7878005	-1.889803208	0.57307919	-3.29763015	0.00097504	0.01418571
SMPD1	806.050582	-1.047229366	0.24361984	-4.29862103	1.72E-05	0.0009255
SMPDL3B	426.136285	-1.587764313	0.33350255	-4.76087604	1.93E-06	0.00018684
SMTN	2591.94602	1.350980989	0.36922288	3.65898506	0.00025322	0.00573303
SNCA	723.855346	1.292661033	0.47410239	2.72654402	0.00640014	0.04843061
SNED1	153.855118	-3.105173408	0.4950927	-6.27190298	3.57E-10	3.69E-07
SNORA61	14.4666716	-1.361858707	0.4894451	-2.78245446	0.00539494	0.04357113
SNORD17	14.5032155	2.157549436	0.62374045	3.45905007	0.00054208	0.00968569
SNTB1	543.521919	-1.968614637	0.6439687	-3.05700363	0.00223562	0.02460764
SOX15	1949.01222	1.511940788	0.28648645	5.27752987	1.31E-07	2.78E-05
SOX4	14107.1345	-1.355166296	0.17856403	-7.58924563	3.22E-14	1.66E-10
SOX5	14.4447851	-2.247868139	0.77865548	-2.88685845	0.00389109	0.03484235
SOX7	3493.89156	1.483679547	0.34873913	4.25441083	2.10E-05	0.00104189
SPNS2	351.729301	-5.45870114	0.90860638	-6.00777329	1.88E-09	1.15E-06
SPRY3	96.6930345	-1.092966913	0.26038496	-4.19750394	2.70E-05	0.00124567
SRD5A3	2036.30783	-1.256514468	0.39029481	-3.21939839	0.0012846	0.01699901
SRGAP2D	98.487889	1.585620638	0.4931666	3.21518253	0.00130362	0.01714478
SRPX	202.955737	1.691421856	0.59074903	2.86318179	0.0041941	0.03642009
STAC	57.1934093	2.344935486	0.60676817	3.86463166	0.00011126	0.00335045
STAC2	26.1015132	-4.0056073	0.86261066	-4.6435866	3.42E-06	0.00028204
STAR	13.6629638	2.034202698	0.62281926	3.26612044	0.00109032	0.0152753
STK31	35.3984767	-2.972069526	1.05575479	-2.81511347	0.004876	0.04041756
STRIP2	417.7782	1.351457342	0.39985177	3.37989587	0.00072513	0.01169032
STX11	61.0889024	-1.231308784	0.45192534	-2.72458451	0.00643825	0.04853945
SUGCT	239.760008	1.735434413	0.51880736	3.34504588	0.00082269	0.01269561
SULT1A2	41.1984666	-2.231186219	0.45433183	-4.91091774	9.07E-07	0.00010899
SULT1E1	83.5330773	-2.223091874	0.60408594	-3.68009204	0.00023315	0.00542117
SUSD2	256.764786	-2.774936963	0.89603868	-3.09689418	0.0019556	0.02241637
SUSD4	166.015608	-2.078521837	0.63857174	-3.25495431	0.00113411	0.01562616
SUV39H1	396.164935	1.036462884	0.26839614	3.86169073	0.00011261	0.00338122
SYBU	873.194971	-1.749968027	0.40410268	-4.33050343	1.49E-05	0.00083596
SYN1	50.8751428	1.340472742	0.46136493	2.90545001	0.00366725	0.03377283
SYPL2	26.7003697	-3.343339135	1.08850185	-3.07150523	0.00212982	0.02376363
SYT17	181.077237	-1.641708576	0.31353742	-5.23608506	1.64E-07	3.22E-05
TACR1	21.8516733	-5.276642109	1.00736629	-5.23805704	1.62E-07	3.22E-05
TAGLN3	34.3433278	2.58847695	0.56397397	4.58970996	4.44E-06	0.00034079
TBC1D30	259.759874	-1.461200681	0.32784281	-4.45701609	8.31E-06	0.00053503
TBX18	33.7220436	1.853171533	0.48109091	3.85201944	0.00011715	0.00344936
TC2N	3797.50393	-1.127341315	0.30799227	-3.66029089	0.00025193	0.00571938
TCF19	781.929015	1.299425067	0.43820206	2.96535589	0.00302333	0.02977068
TENM2	4499.63607	1.543334361	0.56306508	2.74095198	0.00612615	0.04698833
TEPP	22.9174783	2.949181446	0.79658086	3.70230015	0.00021365	0.00512141
TFF3	92.5021469	-1.593438074	0.52795489	-3.01813298	0.00254337	0.02668658
TGFB1	5602.10785	1.192949792	0.29021277	4.11060402	3.95E-05	0.00161484
TGFBI	23621.8529	1.063403575	0.36224122	2.93562279	0.00332879	0.03175042
TGM1	6392.1666	2.211070733	0.56545417	3.91025633	9.22E-05	0.00291817
THSD7A	58.2611587	-4.972869821	1.04401059	-4.76323694	1.91E-06	0.00018583
TIMP1	7275.34186	1.206094723	0.4029527	2.99314216	0.00276121	0.02791631
TINAGL1	22777.1618	1.275105917	0.46080815	2.767108	0.0056556	0.04489242
TJP3	2125.01873	-1.538916022	0.50757012	-3.03192793	0.00242997	0.02591915
TKT	33255.6288	1.333731099	0.28368279	4.70148759	2.58E-06	0.00023153
TLE2	1184.40205	-2.167029258	0.50301574	-4.30807441	1.65E-05	0.00090249
TLN2	169.708812	-1.841958252	0.52585647	-3.50277759	0.00046043	0.0086661

TLR5	458.635914	-1.103717596	0.31220049	-3.53528466	0.00040734	0.00800786
TM7SF2	1223.15975	-1.050215711	0.28254445	-3.71699283	0.00020161	0.00489794
TMC4	3240.78677	-1.012478897	0.31238983	-3.24107514	0.0011908	0.01614691
TMC5	2184.29226	-2.694045445	0.4727301	-5.69890815	1.21E-08	4.45E-06
TMEM116	200.654526	-1.009981306	0.33970927	-2.97307551	0.00294832	0.02934883
TMEM125	744.048555	-1.980641695	0.43643318	-4.53824734	5.67E-06	0.00039988
TMEM132B	25.1985901	-1.695161128	0.50393643	-3.36383919	0.00076866	0.01213971
TMEM139	269.273075	-1.103636243	0.32609465	-3.38440461	0.00071333	0.01161786
TMEM150A	549.060589	-1.074495302	0.21300958	-5.04435193	4.55E-07	6.53E-05
TMEM154	1139.85888	1.261256173	0.38873859	3.24448413	0.00117664	0.01606443
TMEM158	124.380967	1.259242813	0.39553862	3.18361536	0.00145448	0.01838431
TMEM170B	90.3149716	-1.406765749	0.40461803	-3.47677477	0.00050748	0.00917198
TMEM176B	19.9824707	-5.461757987	1.56695328	-3.48559084	0.00049105	0.00900203
TMEM255A	41.8261454	2.340741677	0.61425562	3.81069637	0.00013858	0.00384431
TMEM45A	5054.10336	-2.172986597	0.4356675	-4.98771789	6.11E-07	8.24E-05
TMEM86A	83.7443579	-2.367927167	0.50229412	-4.71422434	2.43E-06	0.00022006
TMPRSS11D	418.03097	2.373366482	0.750582	3.1620349	0.00156671	0.01948522
TMPRSS13	425.175336	-1.791482338	0.44406946	-4.03423902	5.48E-05	0.00206208
TMPRSS3	74.1847813	-4.257765968	0.84662718	-5.02909197	4.93E-07	7.01E-05
TNC	32356.927	1.15407458	0.28616438	4.03290787	5.51E-05	0.00206837
TNFAIP8L1	1016.68187	1.575514693	0.52870163	2.97996944	0.00288277	0.02890039
TNFRSF19	380.562992	-1.27796573	0.43720822	-2.92301397	0.00346661	0.03254458
TNFSF13	242.183643	-1.31473868	0.31730112	-4.14350471	3.42E-05	0.00146538
TNFSF14	47.6023766	-2.287308043	0.70342435	-3.25167597	0.00114727	0.01575994
TNNC1	35.9118245	-2.252537356	0.66143047	-3.40555426	0.0006603	0.01105212
TNRC6C	143.167001	-1.143596356	0.37347262	-3.06206208	0.00219818	0.02426445
TOB2P1	38.0283935	-1.183046631	0.32422955	-3.64879337	0.00026347	0.00590496
TOX3	334.5427	-3.001575077	0.70024245	-4.28647972	1.82E-05	0.00095111
TP53AIP1	150.5595	1.746217972	0.49426726	3.53294282	0.00041096	0.00805384
TP53I11	3926.68006	-1.187689935	0.37928514	-3.1313906	0.00173981	0.02064472
TP53INP2	4139.97861	-1.432406677	0.26213115	-5.46446561	4.64E-08	1.22E-05
TPCN1	2799.39084	-1.034371993	0.25359336	-4.07886067	4.53E-05	0.00179054
TPPP3	25.9514068	-2.806918754	0.79785474	-3.51808244	0.00043468	0.00835367
TPST1	535.120737	1.030423327	0.29628884	3.47776626	0.00050561	0.00914996
TPTE2P6	20.6504606	-2.380846039	0.71751631	-3.31817689	0.00090607	0.01361652
TRIM16L	433.434807	1.09094832	0.29785969	3.66262493	0.00024964	0.00569372
TRIM7	1405.33771	1.024970846	0.29902515	3.42770787	0.0006087	0.01051262
TRND	179.220923	-1.026045889	0.34270081	-2.99399899	0.00275347	0.02787818
TRPV6	110.129545	-3.038098888	0.72254321	-4.20472972	2.61E-05	0.00121741
TSGA10	92.9674118	-1.049789652	0.29670829	-3.53812045	0.00040299	0.00795154
TSNAXIP1	10.8376161	-2.021208379	0.60226563	-3.35600817	0.00079076	0.01232556
TSPAN12	641.647018	-1.621262276	0.36734577	-4.41345019	1.02E-05	0.00062365
TSPAN7	361.298075	1.136406067	0.36468151	3.11616034	0.00183223	0.02144114
TSPAN8	245.482412	-2.138464956	0.65700793	-3.25485411	0.00113451	0.01562616
TTC18	27.7144802	-1.623223393	0.32360653	-5.01604029	5.27E-07	7.44E-05
TTC39A	461.111788	-1.782168492	0.38157761	-4.67052685	3.00E-06	0.0002603
TTC3P1	47.5400504	-1.846847986	0.49297471	-3.74633413	0.00017944	0.00454722
TTC6	58.9589254	-1.318799997	0.41700381	-3.162561	0.00156388	0.01946566
TUBA1B	11100.0874	1.161396043	0.39289408	2.95600294	0.00311654	0.03028474
TUBA4A	5980.13724	1.058223071	0.28651327	3.6934522	0.00022123	0.00524627
TUBB6	10603.1233	1.060058714	0.25784749	4.111185	3.94E-05	0.00161484
TWIST1	106.776341	3.3406998	0.70132006	4.76344539	1.90E-06	0.00018583
TYMS	1564.31141	1.455662262	0.44022131	3.30666018	0.00094415	0.01390121
UBD	145.423503	-3.754578026	0.73029059	-5.14121102	2.73E-07	4.76E-05
UBE2D4	281.193779	-1.093872881	0.22791665	-4.79944266	1.59E-06	0.00016561

UBE2S	2620.70251	1.197041972	0.32345507	3.70079833	0.00021492	0.00513595
UBXN10	110.225521	-2.446320426	0.6087111	-4.01885297	5.85E-05	0.00212911
UCHL1	216.836971	2.720285145	0.6518047	4.17346657	3.00E-05	0.00133698
UHRF1	652.283248	1.086161785	0.36333446	2.98942686	0.00279501	0.02823965
UPK3B	336.279498	-1.286198255	0.47013736	-2.73579249	0.00622303	0.04751989
UPP1	1741.9984	1.052128636	0.29458838	3.57152114	0.00035491	0.00730021
VGLL1	199.270482	-2.085736078	0.76500605	-2.72643083	0.00640234	0.04843061
VIPR1	329.012761	-2.560512755	0.44180252	-5.79560463	6.81E-09	2.79E-06
VMO1	1150.15165	-2.729997345	0.61001855	-4.47526941	7.63E-06	0.00051237
VSIG1	69.8047865	2.026620039	0.40630103	4.98797663	6.10E-07	8.24E-05
VSIG2	480.005814	-1.958937904	0.56237219	-3.48334777	0.00049518	0.00902839
VSTM2L	4064.64666	-1.704906704	0.49099041	-3.47238288	0.00051586	0.00929207
VTCN1	1647.61165	-2.12133921	0.5798495	-3.65843069	0.00025376	0.00573707
WDHD1	763.248529	1.038233318	0.27740205	3.74270236	0.00018205	0.00459844
WDR52	170.897897	-1.580155616	0.31828752	-4.96455412	6.89E-07	8.90E-05
WDR54	492.28786	1.063712299	0.36358361	2.92563321	0.00343756	0.03239882
WEE2-AS1	48.1472992	-1.240492086	0.44200662	-2.80650112	0.00500827	0.04127169
WFDC2	18774.7549	-2.545572416	0.69405657	-3.6676728	0.00024477	0.00560519
WFS1	1006.84016	-1.155235732	0.29133983	-3.96525166	7.33E-05	0.00249363
WNK2	1219.00272	-1.842572968	0.40667136	-4.5308649	5.87E-06	0.00040854
WNT10A	2469.01664	1.036919864	0.37019612	2.80100146	0.00509443	0.0418
XBP1	11506.9636	-1.23864937	0.29555116	-4.19098125	2.78E-05	0.00126695
XRCC2	114.954594	1.504848682	0.42102074	3.57428636	0.00035118	0.00723309
XRCC3	493.401293	1.118582474	0.21742018	5.14479601	2.68E-07	4.72E-05
YPEL1	30.0614112	-2.003323129	0.46715429	-4.28835438	1.80E-05	0.00094808
YPEL2	688.251435	-1.062336491	0.23892974	-4.44622961	8.74E-06	0.00056006
ZBED2	237.55393	3.083314682	0.56183358	5.48795016	4.07E-08	1.09E-05
ZBED3	60.6395238	-1.299863526	0.42518629	-3.05716237	0.00223443	0.02460764
ZBTB46	88.2828541	-1.598746902	0.48304942	-3.30969638	0.00093397	0.01385693
ZDHHC11B	19.46959	-2.276647158	0.56893583	-4.00158868	6.29E-05	0.0022484
ZDHHC23	152.904551	-1.462394499	0.41712952	-3.50585236	0.00045515	0.00860643
ZFHX2	113.287924	-1.344306062	0.40888411	-3.2877435	0.00100994	0.01451858
ZFP3	210.721037	-1.296419853	0.34731322	-3.73271091	0.00018943	0.00472326
ZFP57	89.1639096	-1.779356331	0.61662996	-2.88561447	0.0039065	0.03490201
ZFYVE28	95.3418526	-1.711867071	0.37481466	-4.56723622	4.94E-06	0.00036672
ZMAT1	17.6010222	-2.133172546	0.66991853	-3.18422681	0.00145141	0.01837547
ZMYND12	15.6183478	-1.744492629	0.49485796	-3.5252391	0.0004231	0.0082126
ZMYND15	110.616941	-1.950437225	0.37424212	-5.21169885	1.87E-07	3.54E-05
ZNF117	338.65179	-1.413446813	0.36744944	-3.84664295	0.00011975	0.00348435
ZNF154	75.7938467	-1.161448604	0.30508112	-3.80701567	0.00014065	0.0038815
ZNF367	216.479963	1.439019849	0.40384775	3.56327315	0.00036626	0.00749382
ZNF385D	22.6040431	3.205772369	1.04554252	3.06613295	0.00216847	0.02403916
ZNF492	13.6817489	1.763836173	0.63622969	2.7723261	0.00556572	0.04444842
ZNF552	585.406497	-1.027314988	0.3351599	-3.0651489	0.00217562	0.02408397
ZNF660	13.3518332	-1.21933843	0.4384669	-2.78091327	0.00542062	0.04366266
ZNF662	42.4758111	-1.198856028	0.43775187	-2.7386657	0.00616891	0.04719959
ZNF713	102.545292	-1.12595022	0.31684069	-3.5536793	0.00037988	0.00764661
ZNF732	17.5637325	1.657060735	0.4707329	3.52017194	0.00043127	0.00830872
ZNF763	27.9395582	-1.392095399	0.34991894	-3.97833685	6.94E-05	0.00241583
ZNF815P	35.8145041	-1.361567417	0.34446493	-3.95270254	7.73E-05	0.00260529
ZSCAN16	120.997094	-1.078131262	0.24366288	-4.42468413	9.66E-06	0.000604
ZSWIM5	46.6555416	-1.817963637	0.6005387	-3.02722147	0.00246813	0.02614572

Supplementary table S3: Factors associated with $FHIT^{low}/pHER2^{high}$ signature in NSCLC

		LUAD				LUSC			
		$FHIT^{low}/pHER2^{high}$	Others	OR [95% CI]	*p*	$FHIT^{low}/pHER2^{high}$	Others	OR [95% CI]	*p*
Age	Years	66.0	69.0		0.0198[(a)]	65.0	69.0		0.0004[(a)]
Sex	Male	69 (56.6%)	48 (39.0%)	2.03 [1.22-3.39]	0.0072[(b)]	22 (17.9%)	78 (62.9%)	0.13 [0.07-0.23]	<0.0001[(b)]
	Female	53 (43.4%)	75 (61.0%)			101 (82.1%)	46 (37.1%)		
Tumor size	T1	24 (19.7%)	59 (48.0%)	0.26 [0.14-0.47]	<0.0001[(b)]	15 (12.2%)	48 (38.7%)	0.22 [0.11-0.42]	<0.0001[(b)]
	T2-T4	98 (80.3%)	64 (52.0%)			108 (87.8%)	76 (61.3%)		
Lymph node status	N0	65 (53.7%)	96 (82.0%)	0.25 [0.14-0.46]	<0.0001[(b)]	69 (56.6%)	90 (73.8%)	0.46 [0.27-0.79]	0.0070[(b)]
	N+	56 (46.3%)	21 (18.0%)			53 (43.4%)	32 (26.2%)		
TNM stage	I	50 (41.7%)	87 (72.5%)	0.27 [0.16-0.46]	<0.0001[(b)]	47 (38.5%)	79 (63.7%)	0.36 [0.21-0.60]	<0.0001[(b)]
	II-III	70 (58.3%)	33 (27.5%)			75 (61.5%)	45 (36.3%)		

[a] p-value from the Mann-Whitney test; [b] p-value from the Fisher's exact test
